# Ganglioside GA2-mediated caspase-11 activation drives macrophage pyroptosis aggravating intimal hyperplasia after arterial injury

**DOI:** 10.7150/ijbs.97106

**Published:** 2025-01-01

**Authors:** Yunmin Shi, Tian He, Hong Liu, Xiaodong Li, Zhengxin Li, Qing Wen, Zhaohui Dai, Xuejing Sun, Qian Tan, Wenjing Yang, Youxiang Jiang, Yuanyuan Liu, Hong Yuan, Fang Lei, Yang Yi, Jingjing Cai

**Affiliations:** 1 Department of Cardiology, Third Xiangya Hospital, Central South University, Changsha, 410013, Hunan, China.; 2 Cancer Institute (Key Laboratory of Cancer Prevention and Intervention, China National Ministry of Education), The Second Affiliated Hospital, Zhejiang University School of Medicine, Hangzhou, 310009, China.; 3 Research Center for Life Science and Human Health, Binjiang Institute of Zhejiang University, Hangzhou, 310053, China.; 4 Department of Cardiovascular Medicine, Department of Hypertension, Ruijin Hospital and State Key Laboratory of Medical Genomics, Shanghai Key Laboratory of Hypertension, Shanghai Institute of Hypertension, Shanghai Jiao Tong University School of Medicine, 200020, China.; 5 Department of Cardiology of the Fourth Hospital of Changsha, Changsha Hospital of Hunan Normal University, Changsha, 410006, Hunan, China.; 6 Medical Science Research Center, Zhongnan Hospital of Wuhan University, Wuhan, 430000, Hubei, China.; 7 Department of Cardiovascular Surgery, Ruijin Hospital, Shanghai Jiao Tong University School of Medicine, 200020, Shanghai, China.

## Abstract

Intimal hyperplasia (IH) remains a significant clinical problem, causing vascular intervention failure. This study aimed to elucidate whether gangliosides GA2 accumulated in atherosclerotic mouse aortae and plasma promote the development of IH. We identified that GA2 was remarkably accumulated in both artery and plasma of atherosclerotic patients and mice. Injected GA2 exacerbated IH and mainly co-stained with macrophages after mouse carotid arterial injury model. Intracellular GA2 induced pyroptosis accompanying the IL-1α release, which was blocked by caspase-11 knockout. Mechanistically, GA2 directly activated caspase-4 as a new ligand. And then, activated caspase-4/11 combined and cleaved BID, promoting the cytochrome C release to cytoplasm, which derived gasdermin E-medicated pyroptosis through activation of caspase-9-caspase-3 pathway. Mice transplanted with caspase-11 deficient bone marrow or mice with caspase-11 knockdown in macrophages exhibited an improvement of the IH aggravated by GA2. These findings suggest GA2-mediated caspase-4/11 activation drives macrophage pyroptosis, contributing to IH. Our results provide a potential diagnostic and therapeutic target in IH.

## Introduction

Intimal hyperplasia (IH) remains a significant clinical problem, causing vascular intervention failure. Currently, the plasma low-density lipoprotein and triglycerides of patients with atherosclerosis have been well treated by medication, but vascular remodeling cannot be completely controlled after vascular interventions. Therefore, it is necessary to further explore other potential risk factors and underlying mechanisms of IH. Gangliosides are a family of up to 50 sialic acid-containing glycosphingolipids, which were first found in ganglioside storage diseases 1. Subsequently, gangliosides were found accumulating in the vascular wall of atherosclerotic patients and animal models 2-4. However, whether the gangliosides or specific types of gangliosides contribute to the development of IH remains unknown.

Inflammatory macrophages regulate the migration and proliferation of smooth muscle cells, leukocyte adherence, and the remodeling of extracellular matrix through secreting inflammatory cytokines, such as interleukin-1 (IL-1) and TNF-α, which contribute to the pathology of intimal hyperplasia (IH) 5-10.

Initially, it was found that Murine caspase-11 (Casp11) and its human ortholog Casp4/5 play critical roles in infectious diseases by mediating inflammatory macrophage pyroptosis 11, 12. Subsequently, Casp4/11 involved in noninfectious conditions, such as atherosclerosis, pulmonary arterial hypertension, and nonalcoholic fatty liver disease/Nonalcoholic steatohepatitis, have also been found 13-15. The most important molecular model of Casp4/11 activation is ligand-receptor interaction-mediated activation. Intracellular Lipid A, a lipopolysaccharide (LPS) composition, can be directly detected by Casp4/11. Then, the interaction between lipid A and Casp4/11 triggers Casp4/11 auto-cleavage and subsequent gasdermin D (GSDMD) activation, thereby inducing macrophage pyroptosis 11, 12. Ganglioside GA2 (GA2) is a subclass of gangliosides, also known as asialo ganglioside GM2 (asialo GM2) 1. The structure of GA2 is similar to lipid A as both of them contain a polar glycan headgroup and fatty acid chains 1, 16. However, whether GA2 could be recognized by intracellular Casp4/11 and induce Casp4/11 activation has not been reported.

In this study, we identified that GA2 was remarkably accumulated in both artery and plasma of atherosclerotic patients and mice. Injected GA2 exacerbated IH and was largely expressed in macrophages after mouse carotid arterial injury. GA2 was uptake by mouse peritoneal macrophages through caveolin-1 and triggered the Casp4/11 activation, N-GSDME-mediated pyroptosis, and IL-1α release of macrophages. Knockout Casp11 blocked the macrophage pyroptosis. Mechanistically, we identified that GA2 is directly bound to and activates Casp4. Additionally, we determined that GA2-Casp11-mediated BID-cytochrome C-Casp9-Casp3-GSDME pathway was the critical mechanism in macrophage pyroptosis. Further, we demonstrated that transplantation with Casp11 deficient bone marrow or bone marrow-derived macrophage-specific caspase-11 knockdown significantly protected the IH aggravated by GA2. Taken together, these findings suggest a potential diagnostic and therapeutic strategy for IH.

## Materials and Methods

### Antibody

Primary Antibody were used for immunofluorescent staining as follows: caspase-11 (1:100, MAB86481, R&D Systems); BID (1:50, sc-373939, Santa Cruz); F4/80 (1:100, 27044-1-AP, Proteintech); F4/80 (1:100, ab16911, Abcam); IL-1α (1:50, sc-9983, Santa Cruz); N-GSDME (1:100, 38821S, CST); Ganglioside GA2 (1:100, ab23942, Abcam), CD68 (1:100, ab955, Abcam). Secondary goat anti-mouse (FITC) antibody (1:100, SA00003-1, Proteintech), goat anti-rat (AF647) antibody (1:200, ab150159, Abcam), goat anti-rabbit (Cy3) antibody (1:200, bs-0295G-Cy3, BIOSS), goat anti-rat (Cy3) antibody (1:200, bs-0293G-Cy3, BIOSS), goat anti-rabbit (FITC) antibody (1:200, bs-0296G-FITC, BIOSS) and goat anti-mouse (AF647) antibody (bs-0296G-AF647, BIOSS) were used.

BV421-conjugated anti-mouse CD31 Antibody (1:100, 102423, BioLegend), FITC-conjugated anti-F4/80 (1:100, 123107, BioLegend), APC-conjugated anti-CD45 (1:100, 157605, BioLegend), antibodies against αSMA (1:100, 14-9760-80, eBioscience) and Ganglioside GA2 (1:100, ab23942, Abcam), secondary goat anti-mouse (AF647) antibody (1:200, bs-0296G-AF647, BIOSS) and goat anti-rabbit (PE) antibody (1:200, bs-0295G-PE, BIOSS) were used for the flow cytometry analysis.

The following primary antibodies were used for western blot: MLKL (1:1000, 37333S, CST), Phospho-MLKL (1:1000, 37333S, CST), RIPK3 (1:1000, 95702S, CST), Phospho-RIPK3 (1:1000, 91702S, CST), GSDMD (1:500, sc-393581, Santa Cruz), GSDME (1:1000, ab215191, Abcam), caspase-11 (1:1000, ab180673, Abcam), caspase-1 (1:1000, ab179515, Abcam), caspase-4 (1:1000, M029-3, MBL), caspase-8 (1:1000, 9746T, CST), caspase-9 (1:1000, 9508T, CST), caspase-3 (1:1000, 9662S, CST), cytochrome C (1:1000, 11940S, CST), BID (1:1000, AF860, R&D Systems), BIM (1:500, sc-374358, Santa Cruz), PUMA (1:500, sc-374223, Santa Cruz), COX IV (1:5000, 11242-1-AP, Proteintech), β-actin (1:5000, BS6007M, Bioworld). The HRP-conjugated secondary antibodies used were anti-rabbit, mouse, rat, or goat from the Proteintech (1:5000, SA00001-2, SA00001-1, SA00001-15, and SA00001-4, respectively).

### Mice

All animal experimental protocols were approved by the Institutional Animal Care and Use Committee of Central South University. All animals received humane care according to NIH Guide for the Care and Use of Laboratory Animals. Specific pathogen-free (SPF) C57BL/6J (wild type, WT) mice were purchased from Hunan SJA Laboratory Animal, Co, Ltd. Apolipoprotein E-deficient (APOE^-/-^) mice on C57BL/6J genetic background were purchased from Changzhou Cavens Laboratory Animal Co, Ltd. Casp11^-/-^ mice on C57BL/6J genetic background previously described 14. Mice were bred and maintained in SPF conditions at the Central South University animal facility. Water and food were available ad libitum.

### Atherosclerotic mouse model

Eight weeks male ApoE^-/-^ mice fed a high-fat and high-cholesterol diet (D12108C, Research Diets) for sixteen continuous weeks were established atherosclerotic model. Male WT mice fed a normal laboratory diet for 16 weeks served as controls.

### Bone marrow transplantation model

Bone marrow transplantations were conducted as previously described 17. Briefly, bone marrow was isolated from 6 to 8-week-old WT or Casp11^-/-^ donor mice. For euthanasia, mice were anesthetized with 3% isoflurane by inhalation followed by cervical dislocation. At the age of 10 weeks, recipient mice received a dose of whole-body irradiation (total irradiation dose of 9-Gy) and 4 hours later received 10^7^ bone marrow cells from donor mice. Six weeks later, a carotid artery wire injury was conducted. Immunoblots for analyzing the Casp11 expression of bone marrow-derived myeloid cells from chimeric mice.

### Establishment of a BM MФ specific Casp11-knockdown murine model

Adeno-associated serotype 2/9 virus (AAV2/9) encoding Casp11 shRNA under control of the macrophage-specific promoter (F4/80) (AAV-F4/80-Casp11) and AAV2/9 carrying non-specific sequence served as control (AAV-F4/80-NC) were obtained from Hanbio (Shanghai, China) 18, 19. To establish a bone marrow macrophage (BM MФ) specific Casp11 knockdown murine model, AAV2-F4/80-Casp11 was delivered to C57BL/6J mice through intravascular injection at 7 days after bone marrow transplant. 21 days after injection, a carotid artery injury model was applied. In this study, male or female mice were injected with 60 μL of an AAV2/9-F4/80-Casp11 viral titer at 1.2 × 10^13^ vector genomes (vg)/mL or an AAV2/9-F4/80-NC viral titer at 1.2 × 10^13^ vg/mL.

### Carotid artery injury model

Carotid artery wire injury was induced proximal to the carotid bifurcation as previously described 20. Mice were anesthetized with isoflurane by inhalation (3% isoflurane for induction and 1%-1.5% isoflurane for maintenance). Then, the right carotid artery was exposed by blunt dissection, and a small incision was made between the two ligatures placed around the external carotid artery to allow the introduction of a straight wire (diameter 0.36 mm). The wire was passed toward the aortic arch and withdrawn six times with a rotating motion to denude endothelial cells. Ligation of the external carotid artery was applied after the withdrawal of the guide wire to increase the neointimal area. The skin incision was closed with a single suture. For ganglioside GA2 treatment, mice were treated either with vehicle (DMSO) or ganglioside GA2 (AG-CN2-9009, Adipogen, 0.25 mg/kg body weight each time) through tail intravenous injection one day before surgery and every other day after surgery for 7 days or 2 weeks. The injected dose of GA2 was calculated according to the difference (5.976 μg/mL) between the plasma concentration of GA2 from atherosclerosis and normal mice, considering the coefficient of plasma to body weight (the plasma component constitutes approximately 4-5% of the body weight). The injection dose was 0.25 mg/kg body weight each time. 7 days or 28 days after the injury, carotid arteries were collected. For euthanasia, mice were anesthetized via intraperitoneal pentobarbital sodium (50 mg/kg body weight), followed by terminal cardiac bleed.

### Lipidomic analysis in mouse plasma and aortae homogenate

The plasma and aortae were harvested from ApoE^-/-^ mice fed a high-fat and high-cholesterol diet, and WT mice fed a normal laboratory diet. For euthanasia, mice were anesthetized with intraperitoneal pentobarbital sodium (50 mg/kg body weight), followed by withdrawal of blood from the heart. The plasma was collected and frozen at -80°C. The mouse aortae were cleared of surrounding adipose and connective tissue before being collected and then snap-frozen in liquid nitrogen and stored at -80°C. The lipidomic analysis of aortic samples was conducted by Shanghai Applied Protein Technology Co, Ltd. Specifically, to identify complete lipid ions, 120 mg aortae (from 8-10 C57BL/6J mice for a mixed sample, from 4-6 ApoE^-/-^ mice for a mixed sample) and 200 μL plasma were used as an individual sample.

The sample pretreatment was performed under the following conditions: the aortic sample was added 200 μL water and 20 μL internal standard mixture (^13^C-labeled Cer, LPC, PC, LPE, PE, PI, PS, PA, PG, SM, Chol Ester, DG and TG) and mixed with a vortex, added 800 μL MTBE and mixed with a vortex, added 240 μL precooled methanol and mixed with a vortex, ultrasound 20 minutes (min), placed at room temperature for 30min, centrifuged at 14000 g for 15min at 10°C, collected the supernatant organic phase, dried with nitrogen, added 200 μL 90% isopropanol/acetonitrile solution and mixed with a vortex, centrifuged at 14000 g for 15min at 10°C and collected the supernatant. The supernatant was analyzed with a reverse-phase CSH C18 column (Waters, ACQUITY UPLC CSH C18, 1.7 μm, 2.1 mm × 100 mm) using a UPLC system (Nexera LC-30A, SHIMADZU). Analyses were performed under the following conditions: column at 45°C; flow rate at 300 μL/min; the mobile phase A: acetonitrile aqueous solution (acetonitrile: water = 6:4, v/v); the mobile phase B: acetonitrile isopropanol solution (acetonitrile: isopropanol = 1:9, v/v); the elution gradient procedure: 0-2min, B maintained at 30%; 2-25min, B changed linearly from 30% to 100%; 25-35min, B maintained at 30%. Mass spectrometry analysis was performed on a Q-Exactive Plus (Thermo Scientific) operating in either negative (NEG) or positive (POS) electrospray ionization mode. The analysis conditions were as follows: Positive: heater temperature 300°C, sheath gas flow rate 45 arb, aux gas flow rate 15 arb, sweep gas flow rate 1 arb, spray voltage 3.0 KV, capillary temperature 350°C, S-lens RF level 50%, MS1 scan ranges: 200-1800. Negative: heater temperature 300°C, sheath gas flow rate 45 arb, aux gas flow rate 15 arb, sweep gas flow rate 1 arb, spray voltage 2.5 KV, capillary temperature 350°C, S-lens RF level 60%. MS1 scan ranges: 250-1800.

Lipid species were identified using the LipidSearch software version 4.1 (Thermo Scientific). The main parameters were as follows: precursor tolerance: 5 ppm; product tolerance: 5 ppm; product ion threshold: 5%. After normalizing the data of all lipidomics ions (see Lipidomics concentration.xlsx) with auto-scaling, the principal component analysis was performed for dimension reduction (MetaboAnalyst version 5.0, https://www.metaboanalyst.ca). The volcano plot was analyzed based on class concentration data (see Classes concentration.xlsx).

### Human atherosclerotic diseased arteries and plasma

Aortic arteries were collected from 4 patients with aortic aneurysms or aortic dissection during vascular replacement surgery. Tissue sections with or without atherosclerotic lesions are collected for histological staining. The study, which collected patients' aortic arteries, was approved by the Ruijin Hospital Ethics Review Board. All patients provided written informed consent.

Plasma of peripheral blood was collected from patients with coronary angiography-confirmed coronary heart disease (CHD) in the Third Xiangya Hospital. We excluded patients diagnosed with acute myocardial infarction (STEMI/NSTEMI), pulmonary embolism, malignant neoplasms, autoimmune disorders, acute infections, severe infectious diseases, trauma history, recent surgical interventions, advanced heart failure characterized by left ventricular ejection fraction <20%, diabetes mellitus, hepatic dysfunction (alanine aminotransferase levels >135 U/L), renal impairment (creatinine levels >3.0 mg/dL), or blood-borne infectious diseases such as human immunodeficiency virus/acquired immunodeficiency syndrome and hepatitis B and C, and myocarditis and pericarditis. Age, gender, and disease history matched healthy controls (CTL) were enrolled from the check-up center of the Third Xiangya Hospital. Human sample procedures received ethical approval from the Third Xiangya Hospital. Informed consent was obtained from all patients.

### Histological and morphometric analysis

Four to six 6µm-thick frozen sections from carotid arteries of mice cut 100 µm apart from each other were stained with hematoxylin and eosin-stained. The human thoracic aorta was embedded in paraffin and cut into 6-μm-thick sections. Tissue sections were deparaffinized and hydrated, followed by staining with hematoxylin and eosin-stained or oil-red staining. The 4-6 sections obtained from each arterial segment were analyzed by using ImageJ software (National Institutes of Health, United States), and the average values were calculated. Sections were analyzed separately by two investigators blinded to the study design.

### Immunofluorescent staining

Frozen sections (6 μm) were fixed with 4% paraformaldehyde for 10 min, permeabilized with 0.2% Triton X-100 for 10 min, and blocked with 5% BSA for 60 min at RT. 6-μm-thick paraffin slice of human thoracic aorta was deparaffinized and hydrated followed by antigen retrieval with 98°C water bath for 20 mins. The antigen retrieval solution used was prepared as 10 mM Tris, 1 mM EDTA, and 0.05% Tween-20 (pH 9.0). After cooling down to room temperature (RT), sections were permeabilized with 0.2% Triton X-100 for 10 min and blocked with 5% BSA for 60 min at RT. Samples were then incubated in primary antibodies over night at 4°C followed by incubation for 60 min with fluorescent-labeled secondary antibodies at RT. After nuclear staining for 10 min with DAPI, slides were covered using Gelvatol. Immunofluorescence images were captured and recorded by a fluorescence microscope (ZEISS) with Vert.A.1 software, and the signal intensity of target proteins was calculated by ImageJ software.

### Flow cytometry analysis

Single-cell suspensions isolated from mouse aorta were as previously described 14. For euthanasia, mice were anesthetized with intraperitoneal pentobarbital sodium (50 mg/kg body weight), followed by terminal cardiac bleed. Injured carotid arteries (from 3 mice for a mixed sample) were harvested and digested in an enzyme solution containing papain (2 mg/ml) (P4762, Sigma-Aldrich) in PBS at 37°C for 1h. A single-cell suspension was prepared by passing the aortic pieces through a strainer and subsequently stained for flow cytometry.

For isolation of peripheral blood mononuclear cells (PBMCs), the blood was diluted with Hank's Balanced Salt Solution (Hyclone, Logan, UT, USA) at a 1:1 ratio and then carefully added to Ficoll-Paque solution at a 4:3 ratio in 50 mL conical tubes, followed by centrifugation at 400g for 40 min at 20°C. The PBMC layer was transferred and washed with RPMI-1640 medium, followed by centrifugation at 500 g for 10 min at 4°C. The PBMC pellets were washed again. A single-cell suspension was subsequently stained for flow cytometry.

For isolation of bone marrow cells (BMCs), the tibia and femur of mice were surgically separated, followed by extraction of bone marrow through the flushing of the bone cavity with sterile PBS. A single-cell suspension was prepared by passing a strainer and subsequently stained for flow cytometry.

Cell suspensions were stained with antibodies against the cell surface marker. Then, the intracellular staining was performed according to the manufacturer's manual (GAS003, Invitrogen). Absolute cell counts were detected by flow cytometry (BD FACSCalibur, United States). Cells were sorted using the FACS Aria II (BD) system. Data was analyzed using FlowJo software version X (Tree Star Inc., United States).

### Cell culture

All cells were cultured at 37°C in an atmosphere of 5% CO2. RAW264.7 and HEK293T cell lines obtained from the ATCC were cultured in DMEM (Gibco, NY, USA) containing 10% (v/v) fetal bovine serum (FBS, Gibco), 100 μg/mL streptomycin, and 100 U/mL penicillin.

Peritoneal macrophages (PMs) were isolated from mice. Mice were intraperitoneally injected with 3 mL of sterile 3% thioglycollate to elicit peritoneal macrophages for 72 h. For euthanasia, mice were anesthetized with 3% isoflurane by inhalation, followed by cervical dislocation. Peritoneal macrophages were collected by three rounds of peritoneal cavity lavage using 5 mL of RPMI-1640 medium (Gibco, NY, USA). Peritoneal macrophages were resuspended and cultured in RPMI-1640 medium containing 10% (v/v) fetal bovine serum (Gibco), 100 μg/mL streptomycin, and 100 U/mL penicillin.

Human monocyte-derived macrophages (HMDMs) were isolated from heparinized venous peripheral blood obtained from healthy volunteers in our research group aged 20 to 35 years. The isolation of PBMCs has been previously described. The pellets were then resuspended and cultured in RPMI-1640 medium supplemented with 20% (v/v) fetal bovine serum (Gibco), 100 μg/mL streptomycin, 100 U/mL penicillin, and 50 ng/mL human macrophage colony-stimulating factor (hM-CSF, APExBIO, P1038) for 7 days. The growth medium was replaced with fresh medium 48 h after seeding and then every 2 days. Ethical approval was received from the Third Xiangya Hospital of Central South University ethics committee and adhered to the principles of the Declaration of Helsinki.

### Cell treatment

RAW264.7 cells were primed with 100 ng/mL LPS (tlrl-3pelps, InvivoGen) for 6 h. Then, 6 μg of ganglioside GA2 was transfected using Lipo2000 transfection reagent for 20 h according to the manufacturer's instructions. For LPS-induced pyroptosis, primed cells were transfected with 6 μg of LPS for 16 h. Necroptosis was induced by pretreatment with z-VAD-FMK (20 µM; A1902, ApexBio) and Smac mimetic (10 µM; LCL-161, TargetMol) for 30 min, followed by TNFα (30 ng/mL; HY-P7090, MCE) for 4 h. For mouse peritoneal macrophages and human-monocyte-derived macrophages treatment, cells were pretreated for 6 h, followed by the direct addition of 4 μg/mL GA2 treatment for 24 h.

### Knockout Casp11 by using CRISPR-Cas9

Lentivirus (pHBLV-U6-gRNA-EF1-CAS9-puromycin) expressing Cas9 and sgRNAs targeting Casp11 and control lentivirus were obtained from Hanbio (Shanghai, China). The targeting sequence of the sgRNA used is TTCCTGGTGCTAATGTCTCA for Casp11. Briefly, once the RAW264.7 cells reached approximately 60% confluency in a 6-well plate format, cells were infected with the above lentiviruses (MOI of 50) and 7 μg/mL polybrene (H9268, Sigma). The transfected cells were continuously screened with 2.0 μg/mL puromycin (A1113802, Gibco) for 7 days per cycle. Cells surviving the puromycin screening were expanded and used for further experiments.

### Transfection of siRNA

Raw 264.7 cells, which reached ~40% confluency, were transfected with siRNA to knock down BID according to the manufacturer's instructions for the Lipo3000 transfection reagent (L3000015, Invitrogen). Sixty picomoles of siRNA were used for cells cultured in 6-well plates. A negative control siRNA and siRNAs targeting BID were purchased from Tsingke (Beijing, China). RNAiMAX transfection reagent (13778075, Invitrogen) was used to knock down caveolin-1 expression in PMs according to the manufacturer's instructions. Eighty picomoles of siRNA were used for cells cultured in 6-well plates. Control siRNA and siRNAs targeting caveolin-1 were synthesized by HANBIO (Shanghai China).

### Plasmid transfection

HEK293T cells reached ~60% confluency and were transfected with a plasmid according to the manufacturer's instructions for the Lipo2000 transfection reagent (11668019, Invitrogen). A total of 2.5 μg of plasmid was used for cells cultured in 6-well plates. Plasmids were purchased from Tsingke (Beijing, China) as follows: pCMV-3×Flag-BID 1-195, pcDNA3-Casp4-HA 1-377 C258A (FL), pcDNA3-Casp4-HA 1-289 C258A (p33). Plasmids obtained from Sangon (Shanghai, China) were as follows: pcDNA3-3×Flag-Casp4 1-377, pcDNA3-Casp4-HA 102-289 C258A (p22), pcDNA3-Casp4-HA 290-377 (p10).

### Separation of the cytosol and mitochondria fraction

Mitochondrial and cytosolic fractions from RAW264.7 cells were prepared using the Mitochondria Isolation Kit for Cultured Cells (89874, Thermo Fisher Scientific). Briefly, cells were collected with cold PBS. The collected cells were resuspended in 800 µL Mitochondria Isolation Reagent A (with protease inhibitor) and vortexed at medium speed for 5 seconds before being incubated on ice for exactly 2 minutes. Then the cell suspension was homogenized with a Dounce Tissue Grinder to lyse the cells and was collected in a 2.0 mL microcentrifuge tube. 800 µL of Mitochondria Isolation Reagent C (with protease inhibitor) was added to lyse the cells in the 2.0 mL microcentrifuge tube, followed by centrifugation at 700 g for 10 minutes at 4°C. The supernatant was further centrifuged at 12000 g for 15 minutes at 4°C to pellet the mitochondria. The supernatant containing the cytosol fraction was transferred to a new tube. The pellet containing the isolated mitochondria was washed with 500 µL Mitochondria Isolation Reagent C (with protease inhibitor), followed by centrifugation at 12000 g for 5 minutes at 4°C. The pellet was collected in another tube and used as the mitochondria fraction.

### Cell death analysis

After stimulation, the medium was replaced with 1 mL of Hank's Balanced Salt Solution and incubated with 200 nM SYTOX Green (S7020, Thermo Fisher Scientific) for 30 min. Then, the images were captured and recorded by a fluorescence microscope (ZEISS) using Vert.A.1 software. The resulting images were analyzed using ImageJ software, which counts the number of SYTOX Green-positive cell nuclei present in each image.

### LDH cytotoxicity assay

Cells were treated as indicated. Then, the release of LDH was determined using the LDH Cytotoxicity Assay Kit (Beyotime, China) according to the manufacturer's instructions manual.

### Cytokine measurement

Cells were treated as indicated. The collected supernatant was determined using Mouse IL-1α ELISA Kits (EM011-96, Excell Bio) and IL-1β ELISA Kits (EM001-96, Excell Bio) according to the manufacturer's instructions instructions.

### ELISA assay for testing GA2 levels in plasma

The human plasma was collected from 8 patients with coronary heart disease (CHD) and 8 healthy controls (CTL). Four hundred μL of plasma were used as an individual sample and frozen at -80°C. ELISA assays for testing GA2 levels in plasma were conducted according to the manufacturer's instructions (ab23942, Abcam).

### Pull-down assay

HEK293T cells overexpressing Casp4 or HMDMs were lysed in 0.7 mL lysis buffer containing 50 mM Tris-HCl (pH 7.4), 150 mM NaCl, 10% glycerol, and 1% NP-40, with the addition of a protease inhibitor (B14001, Bimake). 600 μL of lysates were equally divided into 2 tubes, and the remaining lysates were saved as input. 10 μg of ganglioside GA2 or 5 μL of DMSO (as a monitor) were added to the 2 tubes respectively and incubated overnight at 4°C on a nutator. Anti-GA2 antibody (ab23942, Abcam) was used to produce antibody-conjugated beads according to the manufacturer's instructions for protein-A/G magnetic beads (B23201, Bimake). Then, GA2 antibody-conjugated beads were added to all of the tubes and incubated at 4°C overnight. The beads were washed 3 times with 1 mL of lysis buffer. 60 μL of 1xSDS loading buffer was used to resuspend the beads and heated at 95°C for 10 min. Eluted protein complexes were detected by western blot analysis.

### Caspase activity assay

To measure ligand directly inducing Casp4 activation, each ligand (5 μg) was incubated with 0.125 μM Casp4 proteins (TP760359, Origene) in a 100 μL reaction buffer containing 50 mM HEPES (pH 7.5), 150 mM NaCl, 3 mM EDTA, and 0.005% (v/v) Tween-20 and 10 mM DTT at 37 °C for 30 min. After incubation, a fluorogenic substrate zVAD-AMC (I-1710.0005, BACHEM) was added to the reaction at a final concentration of 80 μM for 30 min. Casp4 activity was detected at ex365 nm/em450 nm on a fluorescent multi-well reader (PerkinElmer EnSpire Multimode Plate Reader).

### Co-immunoprecipitation

HEK293T cells overexpressing Casp4 and BID were lysed in 0.5 mL lysis buffer (P0013, Beyotime) containing a protease inhibitor. A total of 2000 μg of protein was used for the co-immunoprecipitation assay. The remaining protein was taken out and saved as input. Lysates containing 2000 μg of protein and 30 μL of anti-Flag magnetic beads (B26102, Bimake) were incubated overnight at 4°C on a nutator. The beads were washed 3 times with 1 mL of lysis buffer. 60 μL of 1x SDS loading buffer was used to resuspend the beads, followed by heating at 95°C for 10 minutes. Eluted protein complexes were detected by western blot analysis.

### Real-time quantitative PCR

Total RNA was isolated from PBMCs and BMCs using TRIzol reagent (15596-026, Invitrogen). First-strand cDNA was synthesized from 3 μg of RNA using the cDNA Synthesis Kit (Thermo Fisher, K1691). PCR amplification was performed with SYBR Green PCR Master Mix (1725214, BioRad, Hercules, CA, United States). The primer sequences of the primers used in this study were as follows: mouse CASP4 (forward: CCGGAAACATGCTTGCTCT and reverse: TCTCGTCAAGGTTGCCCGAT) and mouse ACTB (forward: GTGCTATGTTGCTCTAGACTTCG and reverse: ATGCCACAGGATTCCATACC).

### Western blot

The lysed cell was prepared in RIPA buffer (P0013B, Beyotime) containing 1% protease inhibitor and 1% phosphatase inhibitor. Lysates were cleared by centrifugation, and equal amounts of proteins were separated by SDS-PAGE and immunoblotted with indicated antibodies. The immunoreactivity was visualized and imaged using ECL Plus Kit (K-12045-D50, Advansta) and ChemiDoc™ Imaging System (Bio-Rad, California, USA), and densitometric analysis was performed with Image Lab software (Bio-Rad).

### Statistical analysis

**Figure 1:**
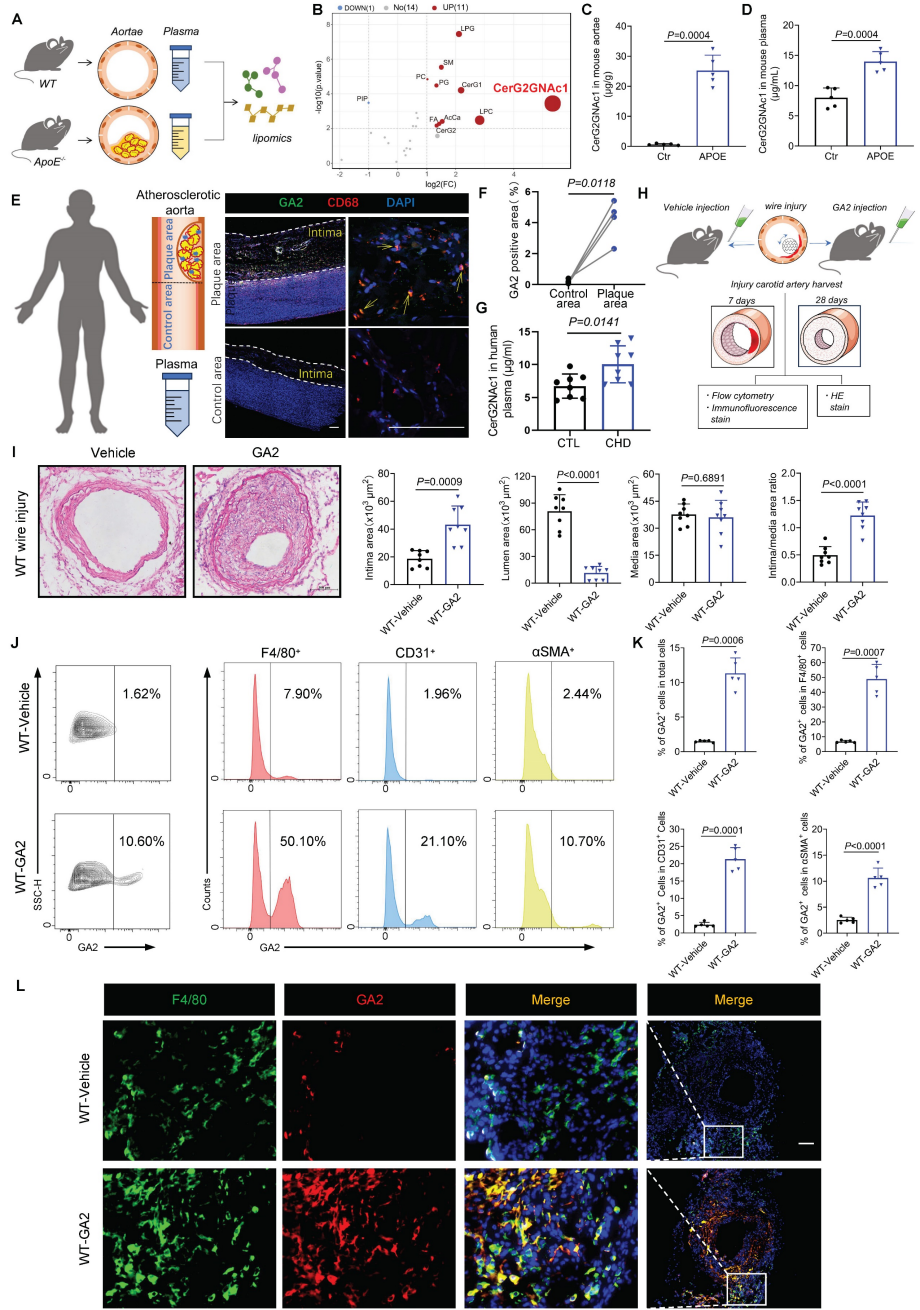
** GA2 remarkably accumulates in both artery and plasma of atherosclerotic patients and mice; and GA2 largely co-expresses with macrophages in carotid arterial injury model aggravating IH. (A)** Experimental design for lipidomics. Wild type (WT) mice fed a normal laboratory diet for 16 weeks served as control (Ctr) group. APOE^-/-^ mice fed high-fat high-cholesterol diet for 16 weeks served as APOE group. **(B)** Volcano plot of the quantified Classes concentration from Ctr and APOE mouse aortae (n = 5). Cut-off criteria of -log10(p.value) > 2 and |log2 fold change (FC)| > 1 were used, APOE vs Ctr.** (C)** The quantified CerG2GNAc1 (ganglioside GA2, GA2) from Ctr and APOE mouse aortae (n = 5). **(D)** The quantified CerG2GNAc1 from Ctr and APOE mouse plasma (n = 5). **(E)** (left) Experimental design for human vascular tissue and peripheral blood, (right) representative immunofluorescence staining sections from human atherosclerotic thoracic aorta for CD68 (red) and GA2 (green) (n = 4). Nuclei were stained with DAPI (blue). Scale bars: 200 μm.** (F)** Quantitative analysis of immunofluorescence staining sections from human thoracic atherosclerotic aorta for GA2 (green) (n = 4). **(G)** The quantified GA2 from plasma of patients with confirmed coronary heart disease (CHD) or patients without CHD (CTR) (n = 8). **(H)** Experimental design for carotid artery injury model. **(I)** Representative photomicrographs of hematoxylin and eosin-stained (HE) sections from WT mice after 28 days (d) of the injury and analysis results of intima area, lumen area, media area and intima/media area ratio (n = 8). WT mice were performed carotid arterial injury model, and treated either with vehicle or GA2 through tail intravenous injection one day before surgery and every other day after surgery for 2 weeks. Scale bars: 100 μm. **(J-K)** Flow cytometry analysis of GA2^+^ cells, GA2^+^ cells within total F4/80^+^ cells, GA2^+^ cells within total CD31^+^ cells and GA2^+^ cells within total αSMA^+^ cells (n = 5). Single-cell suspensions isolated from the injured carotid arteries of mice. WT mice were treated either with vehicle or GA2 through tail intravenous injection one day before surgery and every other day after surgery for 7 d. **(L)** Representative immunofluorescence staining sections from the carotid arteries of mice after 7 d of the injury for F4/80 (green) and GA2 (red) (n = 7). Nuclei were stained with DAPI (blue). Scale bars: 100 μm. **C**, **D**, **I** (Intima and Lumen) and **K** (GA2^+^ in total, F4/80^+^ and CD31^+^) were tested using a two-tailed Welch's *t*-test; **G**,** I** (Media and Intima/media) and **K** (GA2^+^ in αSMA) were tested using a two-tailed Student's *t*-test; **F** was tested using a two-tailed paired *t*-test.

**Figure 2:**
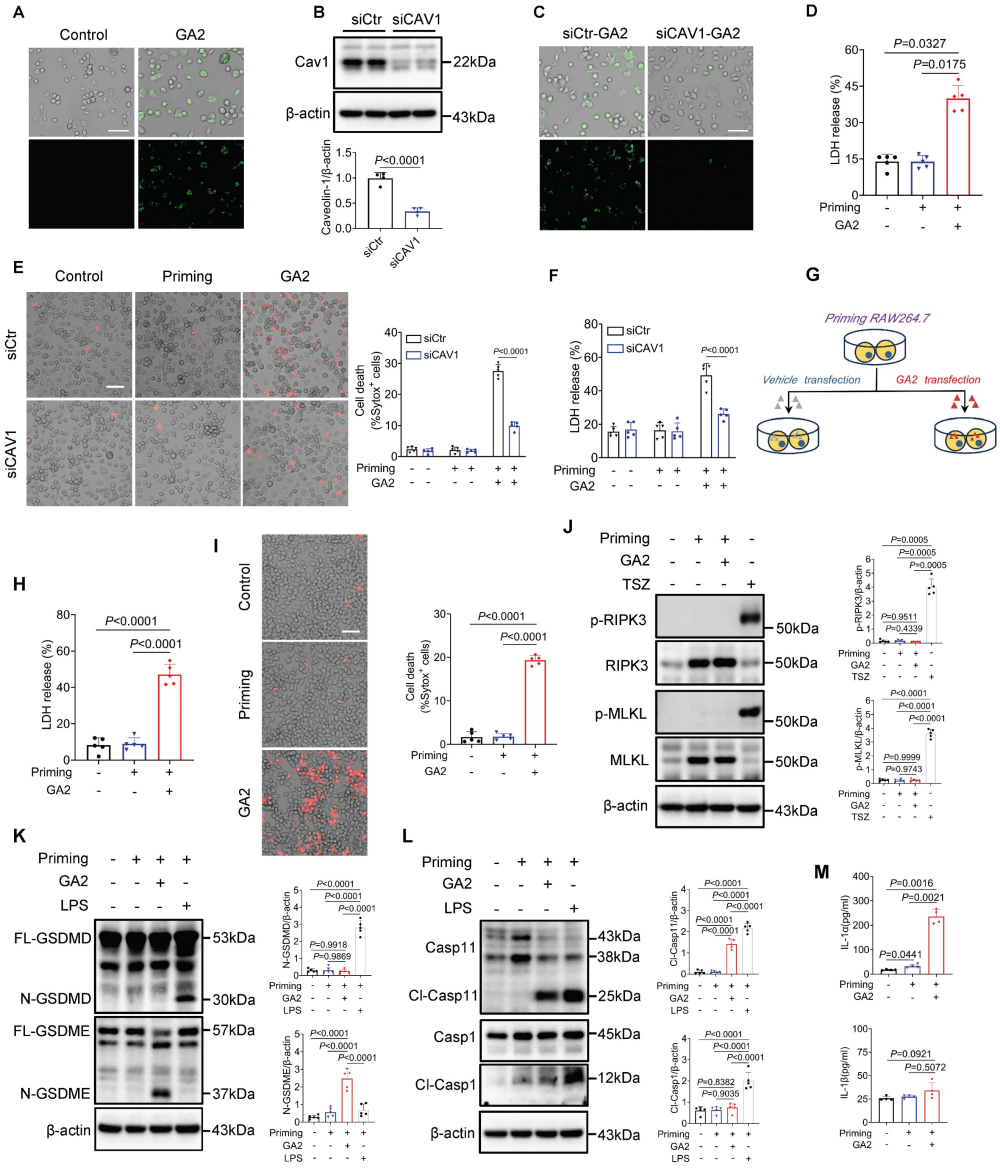
** Caveolin-1 is required for the GA2 uptake in macrophages; and delivers extracellular GA2 into the cytosol induces the activation of Casp11 and GSDME, cell death and the release of IL-1α in inflammatory macrophages. (A)** The uptake of GA2 by mouse peritoneal macrophages (PMs) (n = 6). Green indicates the GA2. PMs were treated with 4 μg/mL fluorescent probe labeled-GA2 for 8 hours (h). Scale bars: 50 μm. **(B)** Immunoblots for analyzeing the knock-down of caveolin-1 (n = 4). PMs were transfected with siCAV1 (siRNAs targeting caveolin-1) or siCtr (Control siRNA) for 48 h. **(C)** The uptake of GA2 by PMs (n = 6). PMs were transfected with siCAV1 or siCtr for 48 h, and then treated with 4 μg/mL fluorescent probe labeled-GA2 for 8 h. Scale bars: 50 μm. **(D)** LDH release of PMs (n = 5). PMs were primed with 100 ng/mL LPS for 6 h to induce inflammatory macrophages. Priming cells were directly stimulated with 4 μg/mL GA2 for 24 h. Priming or non-priming cells stimulated with equimolar DMSO were set as a priming group or a control group. **(E)** Cell death in PMs measured by SYTOX Green uptake assay (n = 5). Red indicates dead cells. PMs were transfected with siCAV1 or siCtr for 48 h, and then treated as as indicated. Scale bars: 50 μm. **(F)** LDH release of PMs (n = 5). **(G)** Experimental design for transfection with GA2 in RAW264.7 cells.** (H)** LDH release of RAW264.7 cells (n = 5). Priming cells were transfected with 6 μg GA2 for 20 h. Priming or non-priming cells transfected with equimolar DMSO were set as a priming group or a control group. **(I)** Cell death in RAW264.7 cells (n = 5). Red indicates dead cells. Scale bars: 50 μm. **(J)** Representative immunoblots of MLKL, pMLKL, RIPK3 and pRIPK3 in RAW264.7 cell lysates and analysis results (n = 5). Cells were triggerd by pretreatment with 20 µM z-VAD-FMK and 10 µM Smac mimetic for 30 minutes (min) and followed by 30 ng/mL TNFα for 4 h (TSZ) to induce necroptosis as a positive control. **(K)** Representative immunoblots of GSDMD, N-GSDMD, GSDME and N-GSDME in RAW264.7 cell lysates and analysis results (n = 5). Priming cells were transfected with 6 μg LPS for 16 h (LPS) to induce pyroptosis as a positive control. **(L)** Representative immunoblots of Casp11, Cl-Casp11 (cleaved-Casp11), Casp1 and Cl-Casp1 in RAW264.7 cell lysates and analysis results (n = 5). **(M)** IL-1α secretion and IL-1β secretion of RAW264.7 cells (n = 4). **B** was tested using a two-tailed Student's *t*-test; **E**, **F**, **H**, **I**, **K**, and **L** were tested using a One-way ANOVA test; **J** and **M** (IL-1α) were tested using a Welch's ANOVA test; **D** and **M** (IL-1β) were tested using a Kruskal Wallis test.

**Figure 3:**
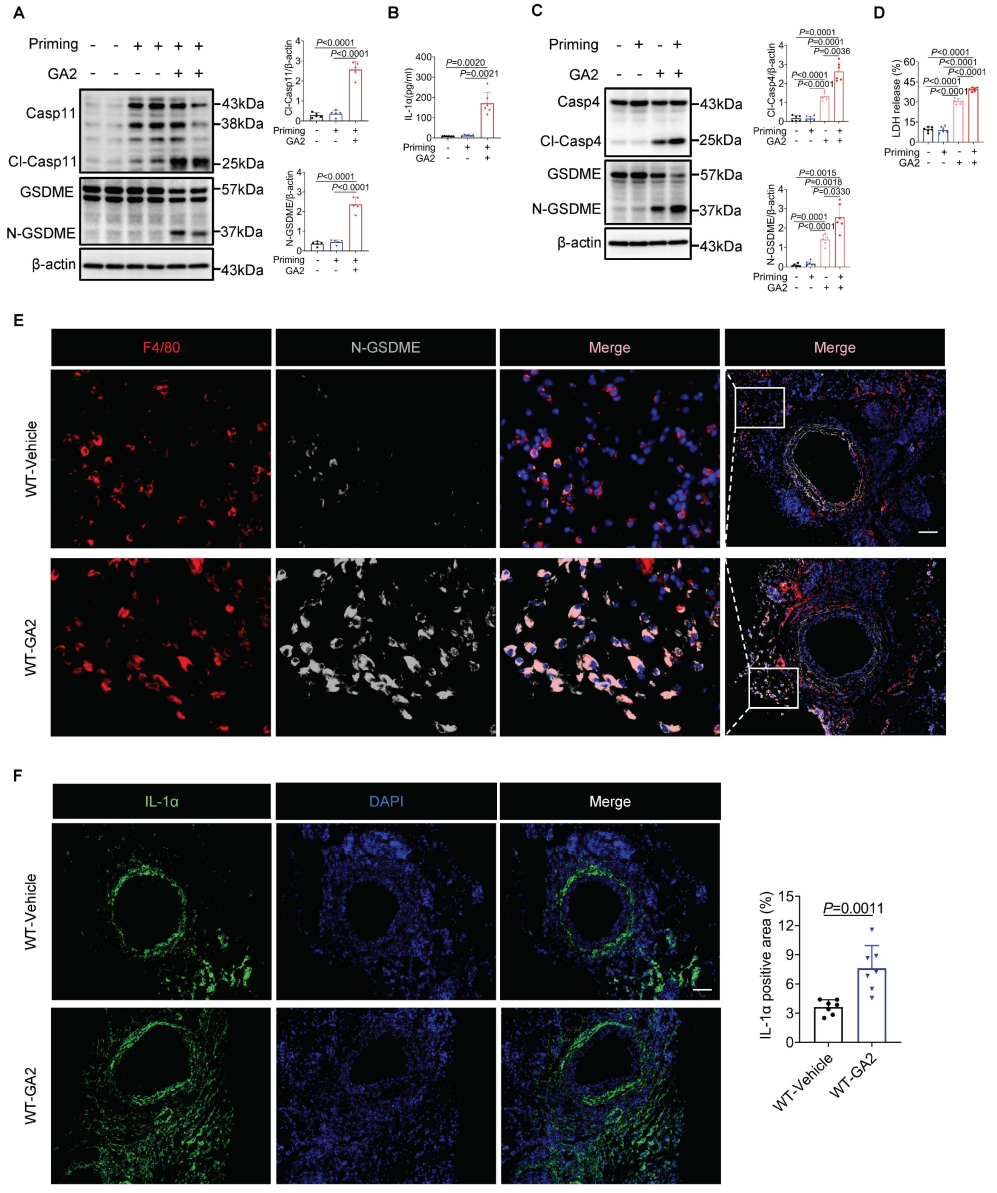
** GA2 triggers primary macrophage pyroptosis *in vitro*, and induces macrophage pyroptosis at injured carotid artery *in vivo*. (A)** Representative immunoblots of Casp11, Cl-Casp11, GSDME and N-GSDME in PMs and analysis results (n = 5). **(B)** IL-1α secretion of PMs (n = 6). **(C)** Representative immunoblots of Casp4, Cl-Casp4, GSDME and N-GSDME in HMDMs and analysis results (n = 6). Priming or non-priming cells were directly stimulated with 4 μg/mL GA2 for 24 h. Priming or non-priming cells stimulated with equimolar DMSO were set as a priming group or control group. **(D)** LDH release of HMDMs (n = 6). **(E)** Representative immunofluorescence staining sections from mice after 7 d of the injury for F4/80 (red) and N-GSDME (light gray) (n = 7). Nuclei were stained with DAPI (blue). Scale bars: 100 μm. **(F)** Representative immunofluorescence staining sections from mice after 7 d of the injury for IL-1α (green) and quantitative analysis. Nuclei were stained with DAPI (blue) (n = 7). Scale bars: 100 μm. **A** and** D** were tested using a One-way ANOVA test; **B** and **C** were tested using a Welch's ANOVA test; **F** was tested using a two-tailed Student's *t*-test.

All data are presented as means ± standard deviation (SD). Numbers (n/N) refer to the number of patients or mice used in experiments - never a technical replicate or an individual experimental replicate performed on a different day, or cells from different individuals. A Shapiro-Wilk test was performed to confirm that the data were normally distributed. When the data met the normal distribution, a two-tailed unpaired Student's t-test was used for equal variance and Welch's t-test for unequal variance (Figure 1C, 1D, 1G, 1I, 1K, 2B, 3F, 8E, 8G, 8I, 8L, and 8N). A two-tailed paired t-test was used for the plaque region and the region without plaque from the same individual (Figure 1F, S1D, and S1E). The nonparametric Mann-Whitney U test was used for the non-normal distribution data (Figure 8G). One-way ANOVA with Tukey's multiple-comparisons test was used for equal variance, and Welch's ANOVA with Dunnett's multiple-comparisons test was used for unequal variance between ≥ 3 groups (Figure 2E, 2F, 2H-2M, 3A-3D, 4A-4H, 5D, 5E, 5G, 5H, 6A-6C, 6F, 6G, 7A-7G, S3A, S3B, S4A, and S4B). If the normality test failed, the nonparametric Kruskal-Wallis test with Dunn's multiple-comparisons test was used (Figure 2D, 2M, and S3A). P < 0.05 was considered a significant finding. Statistical analyses were performed using GraphPad Prism, version 9.4.0.

## Results

### GA2 remarkably accumulates in mouse atherosclerotic aortae and plasma, and GA2 aggravates IH and largely expresses in macrophages after carotid arterial injury

To determine which subtype of gangliosides is remarkably changed in atherosclerotic aortae, a lipomic analysis of mouse atherosclerotic aortae and normal aortae was performed (Figure 1A). A total of 897 lipid species across 26 lipid classes were identified in mouse aortae by lipomic analysis (Figure S1A) (details in Lipidomics concentration.xlsx). As shown in principal component analysis (PCA), lipid species in the aortae of the two groups were significantly separated (Figure S1B). Volcano plot results revealed that 10 up-regulated lipid classes and 1 down-regulated lipid class were identified, with the largest fold change observed in CerG2GNAc1 (Figure 1B). CerG2GNAc1 accumulated in atherosclerotic aortae reached 25.213 μg/g, compared to normal aortae (0.650 μg/g) (Figure 1C) (details in Classes concentration.xlsx). CerG2GNAc1 also significantly accumulated in mouse atherosclerotic plasma, reaching 13.973 μg/mL, compared to normal plasma (7.997 μg/mL) (Figure 1D) (details in CerG2GNAc1 concentration in mouse plasma.xlsx). Moreover, CerG2GNAc1 was identified as ganglioside GA2 (GA2) based on HMDB database comparison and glycosphingolipid nomenclature principle 1. To investigate whether GA2 was upregulated in human atherosclerotic samples, the plaques of patients with thoracic aortic aneurysm or aortic dissection and the plasma of patients with coronary heart disease (CHD) were collected (Figure 1E). Oil-red staining and hematoxylin and eosin staining were applied to confirm the plaque region and the region without plaque (Figure S1C-S1E). A large number of GA2 engulfed by macrophages was observed in the atherosclerotic plaque of the human thoracic aorta (Figure 1E and 1F). Eight patients with angiographically confirmed coronary heart disease (CHD) and eight healthy controls were included. The demographic characteristics of the individuals were summarized in Table S1. The age, sex, white blood cells, liver and renal functions, lipid profile, medication, and comorbidities were comparable between individuals with CHD and healthy controls. However, GA2 levels in individuals with CHD were higher than those in healthy controls (CHD vs. CTL, 9.57 ± 2.64 vs. 5.96 ± 1.43, μg/mL, p = 0.0141) (Figure 1G). To determine the contribution of GA2 to IH, we conducted a carotid artery injury model in wild-type (WT) mice, and the mice were then injected with GA2 (Figure 1H). Surprisingly, the stimulation of GA2 markedly aggravated IH at 28 days after carotid artery injury, indicating an increase in the intimal area and the intima/media area ratio as well as a reduction in the lumen area (Figure 1I). We then performed flow cytometry analysis to test the accumulation of GA2 in various cell types in injured arteries. The flow cytometry analysis showed that GA2 was primarily expressed in macrophages (Figure 1J and 1K). Meanwhile, immunofluorescence staining also showed a largely co-staining of GA2 with macrophages (Figure 1L).

### GA2 is uptake through caveolin-1 by macrophages and triggers macrophage pyroptosis

*In vitro*, we further explored the mechanism of GA2 uptake by macrophages. We observed that GA2 was internalized by PMs *in vitro* (Figure 2A). It has been reported that glycosphingolipids were taken up by cells dependent on caveolin-1-mediated phagocytosis 21, 22. Therefore, we observed that knockdown of caveolin-1 significantly inhibited the internalization of GA2 in PMs (Figures 2B and 2C). Inflammatory macrophages play a crucial role in IH. We further examined the effect of GA2 on the cytotoxicity of inflammatory macrophages. LDH release significantly increased in inflammatory PMs (priming cells) treated with GA2 (Figure 2D). The knockdown of caveolin-1 significantly reduced the number of dead cells and the LDH release (Figures 2E and 2F). Furthermore, we tested whether RAW264.7 macrophages could phagocytose GA2. Differently, only partial GA2 was localized at the intracellularly inflammatory RAW264.7 cells (Figure S2). The lack of caveolin-1 expression in RAW264.7 cells may cause less internalization of GA2 in RAW264.7 cells 23. A transfected system was used to determine the maximum effect of intracellular GA2 in RAW264.7 cells (Figure 2G). The LDH release and the number of dead cells also remarkably increased in inflammatory RAW264.7 cells transfected with GA2 (Figure 2H and 2I).

Necroptosis and pyroptosis are the main forms of programmed cell death induced by plasma membrane rupture. Hence, we examined the critical molecular markers of necroptosis: pMLKL (phosphorylated mixed lineage kinase domain-like protein), pRIPK3 (phosphorylated receptor-interacting protein kinase-3), and the main executioners of pyroptosis: GSDMD and GSDME 24-27. Interestingly, GA2 treatment did not influence the expression of pMLKL and pRIPK3 (Figure 2J) but markedly induced cleavage of GSDME, not GSDMD (Figure 2K) in RAW264.7 cells. Notably, the cleaved Casp11 (Cl-Casp11) was observed, but the cleaved Casp1 (Cl-Casp1) was not (Figure 2L). Unlike Cl-Casp1 processing IL-1β/18, Cl-Casp11 selectively processes IL-1α into mature 28. The secretion of IL-1α, not IL-1β, enhanced significantly in the supernatant of RAW264.7 cells (Figure 2M), indirectly confirming the activation of Casp11. We also observed that GA2 direct stimulation triggered pyroptosis in the inflammatory PMs, including the activation of Casp11 and GSDME, which increased LDH release and IL-1α secretion (Figure 2D, Figures 3A and 3B). Unlike the constitutive expression of human Casp4, Casp11 is expressed at low levels in resting mouse macrophages 28. We further tested the effects on human-derived macrophages and resting mouse macrophages (without priming).

As expected, we observed that direct stimulation with GA2 caused the pyroptosis of HMDMs, including both active and resting states of the cells (Figures 3C and 3D). However, GA2 did not induce significant pyroptosis in resting mouse RAW264.7 cells (Figure S3). These results support that GA2 triggered the GSDME and Casp4/11 activation and pyroptosis in both mouse-derived and human-derived macrophages. Further, we used immunofluorescence staining to test the expression of pyroptosis marker (N-GSDME) with macrophages (F4/80) and pyroptosis-related cytokine (IL-1α) in injured carotid arteries with and without GA2 stimulation. As shown in Figures 3E and 3F, GA2 exposure significantly increased the expression of N-GSDME in macrophages and the expression area of IL-1α. These supported the idea that GA2 mediated macrophage pyroptosis in the arterial injury of mice.

### Casp11 deletion protects GA2-induced macrophage pyroptosis

**Figure 4:**
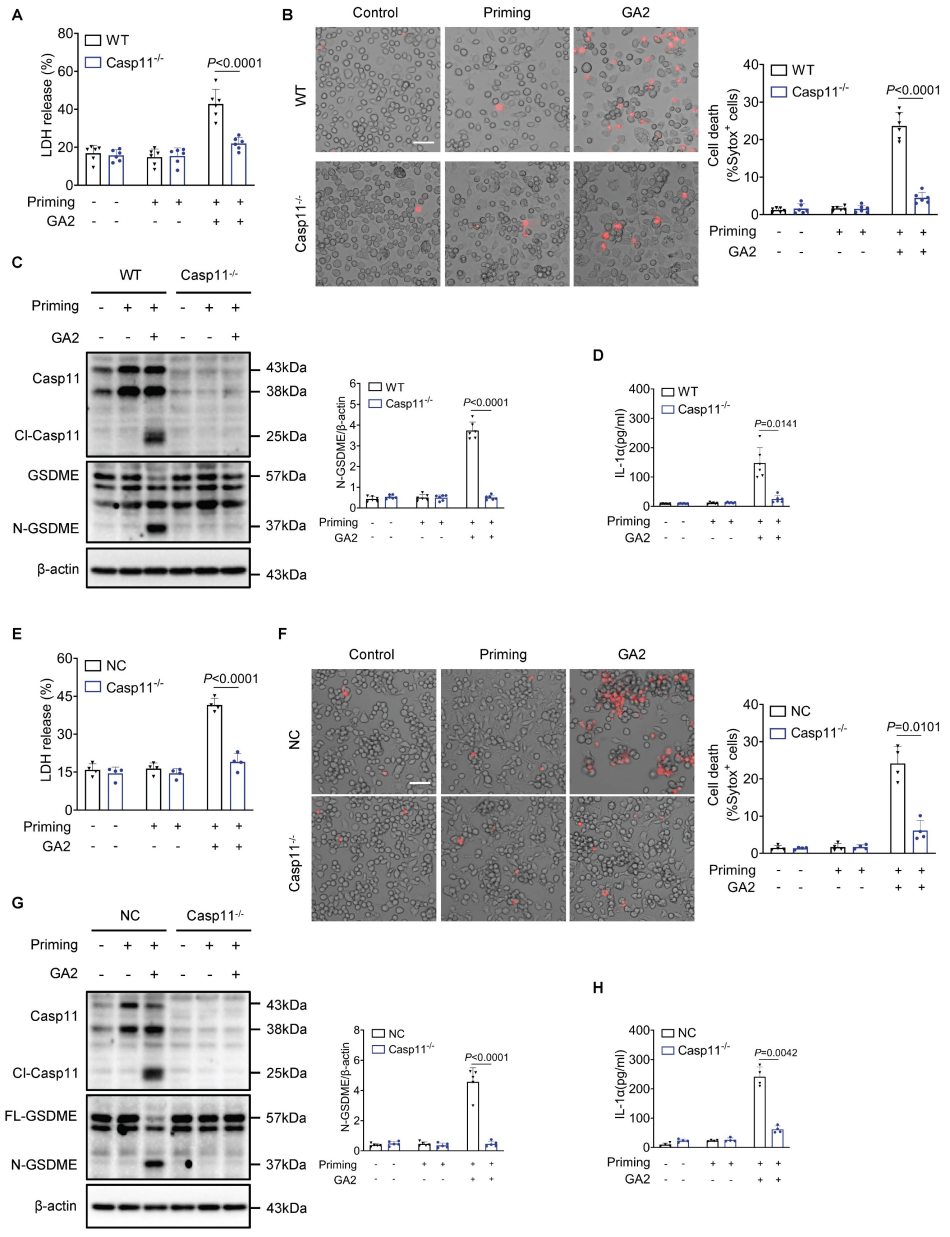
** Deletion of Casp11 blocks GA2-mediated GSDME activation and pyroptosis in macrophages. (A-B)** LDH release** (A)** and cell death** (B)** of PMs (n = 6). PMs derived from Casp11^-/-^ and WT mice were treated as indicated. Scale bars: 50 μm. **(C)** Representative immunoblots of Casp11, Cl-Casp11, GSDME and N-GSDME in PMs and analysis results (n = 6). **(D)** IL-1α secretion of PMs (n = 6). **(E-F)** LDH release **(E)** and cell death **(F)** of RAW264.7 cells (n = 4). Casp11^-/-^ and NC (non-targeted control) RAW264.7 cells were treated as indicated. Scale bars: 50 μm. **(G)** Representative immunoblots of Casp11, Cl-Casp11, GSDME and N-GSDME in RAW264.7 cells and analysis results (n = 5). **(H)** IL-1α secretion of RAW264.7 cells (n = 4). **A**, **E** and** G** were tested using a One-way ANOVA test; **B**, **C**,** D**, **F** and **H** were tested using a Welch's ANOVA test.

Furthermore, we investigated whether Casp11 played a role in regulating macrophage pyroptosis induced by GA2. The deletion of Casp11 remarkably attenuated LDH release and the number of dead cells in PMs (Figures 4A and 4B). Meanwhile, GA2-mediated GSDME activation and release of IL-1α were blocked by Casp11 deficiency (Figures 4C and 4D). These findings were also confirmed in RAW264.7 cells, where Casp11 deficiency significantly inhibited LDH release and the number of dead cells, GSDME activation, and IL-1α secretion (Figure 4E-4H). Taken together, these results indicated that GA2-triggered macrophage pyroptosis depended on Casp11.

### GA2 directly binds to and activates Casp4 in macrophages

**Figure 5:**
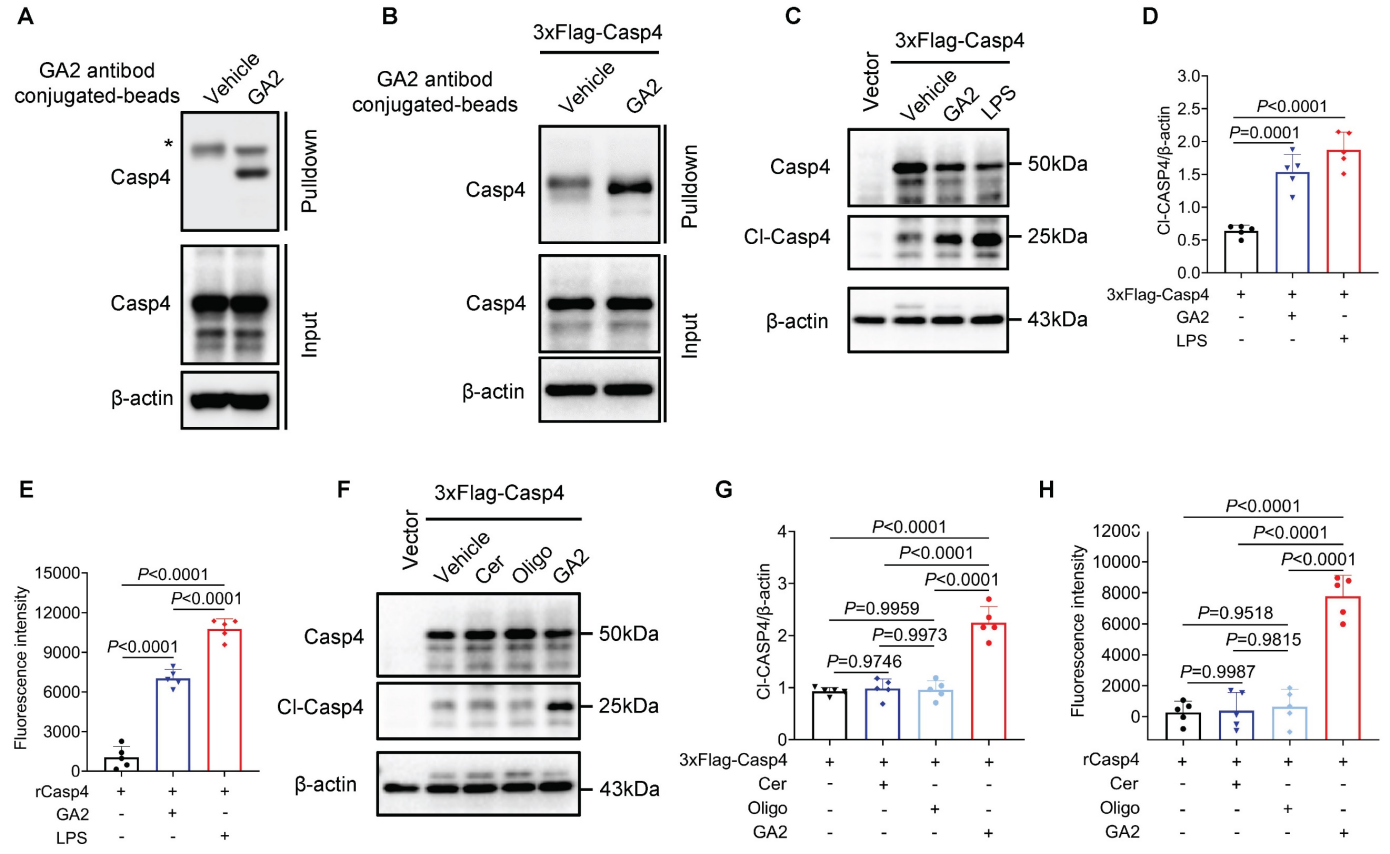
** GA2 binds to and activates Casp4. (A)** Pull-down analysis the interaction between GA2 and Casp4 (n = 3). Lysates from HMDMs were incubated with GA2 or equimolar DMSO (vehicle) and then incubated with anti-GA2 antibody conjugated-magnetic-beads. Casp4 retained by the ligands and inputs were immunoblotted with antibodies against Casp4**. (B)** Pull-down analysis the interaction between GA2 and Casp4 (n = 3). Lysates from HEK293T cells overexpressing 3×Flag-Casp4 were incubated with GA2 or equimolar DMSO (vehicle) and then incubated with anti-GA2 antibody conjugated-magnetic-beads. Casp4 retained by the ligands and inputs were immunoblotted with antibodies against Casp4. **(C-D)** Representative immunoblots of Casp4 and Cl-Casp4 in HEK293T cell lysates and analysis results (n = 5). HEK293T cells transfected with 3×Flag-Casp4 for 40 h, and then transfected with 5 μg/mL GA2, 5 μg/mL LPS and equimolar DMSO (vehicle) for 10 h respectively. **(E)** Activities of recombinant Casp4 (n = 5). 5 μg GA2, 5 μg LPS or equimolar DMSO (vehicle) were incubated with 0.125 μM recombinant Casp4 proteins in a 100 μL reaction buffer for 30 min at room temperature, and then incubated with zVAD-AMC at a final concentration of 80 μM for 30min. **(F-G)** Representative immunoblots of Casp4 and Cl-Casp4 in HEK293T cell lysates and analysis results (n = 5). HEK293T cells transfected with 3×Flag-Casp4 for 40 h, and then transfected with 5 μg/mL ceramide residue (Cer), 5 μg/mL oligosaccharide headgroup (Oligo), 5 μg/mL GA2 or equimolar DMSO (vehicle) for 10 h respectively. **(H)** Activities of recombinant Casp4 (n = 5). 5 μg Cer, 5 μg Oligo, 5 μg GA2 or equimolar DMSO (vehicle) were used. **D**, **E**,** G** and** H** were tested using a One-way ANOVA test.

To further evaluate whether Casp4 (Casp4, Casp11 human ortholog) senses GA2 and initiates Casp4 auto-activation. First, we found the direct binding of Casp4 and GA2 in HMDMs by pulldown assay (Figure 5A). As revealed in Figure 5B, the pull-down assay confirmed the interaction between GA2 and over-expressed Casp4 in HEK293T cells. GA2 transfection significantly increased the expression of cleaved-Casp4 (Cl-Casp4) in HEK293T cells (Figure 5C and 5D). To detect the Casp4 activity, a cell-free system was used. Compared with the vehicle, the incubation of GA2 with recombinant Casp4 triggered substrate Z-VAD-AMC cleaved to emit strong fluorescent intensity (Figure 5E). To determine which group of GA2 is crucial for Casp4 activation, GalNAcβ1-4Galβ1-4Glc (oligosaccharide headgroup, Oligo) and ceramides (ceramide residue, Cer) were used. The transfection of Cer or Oligo did not increase the Cl-Casp4 in HEK293T cells, compared with GA2 (Figure 5F and 5G. Importantly, caspase activity assays confirmed that Cer or Oligo did not activate Casp4 (Figure 5H). These results indicated that GA2 directly bound to and activated Casp4, and the oligosaccharide headgroup and ceramide residue were indispensable.

### Activated Casp4/11 binds to and activates BID leading to cytochrome C-Casp9-Casp3-GSDME pathway activation

**Figure 6:**
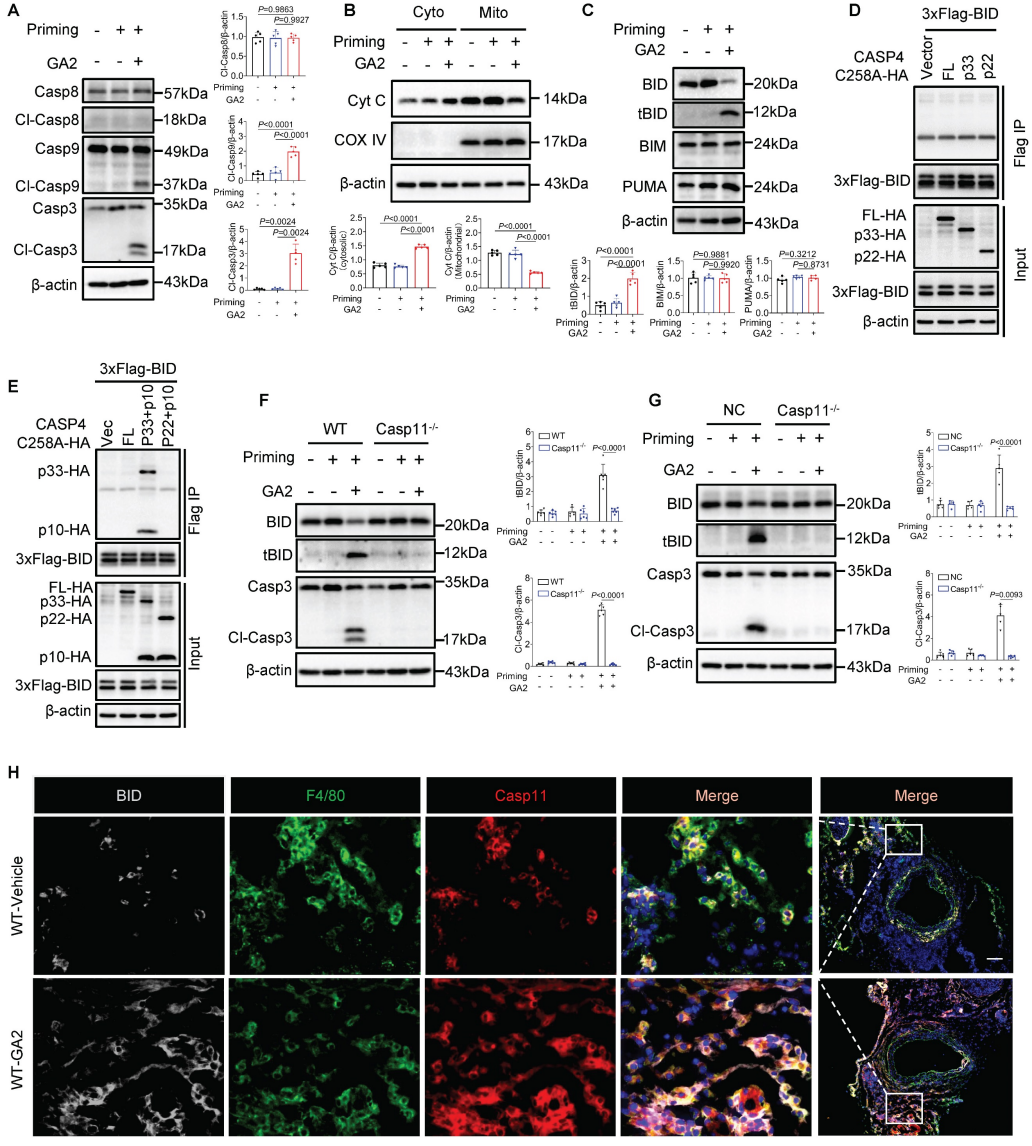
** GA2 mediates Cytochrome C-Casp9-Casp3 pathway activation through Cl-Casp4/11 binding to and cleaving BID. (A)** Representative immunoblots of Casp8, Cl-Casp8, Casp9, Cl-Casp9, Casp3 and Cl-Casp3 in PMs and analysis results (n = 5). **(B)** Representative immunoblots of cytochrome C (Cyt C) in PM cytosol (Cyto) and mitochondrion (Mito) and analysis results (n = 5). **(C)** Representative immunoblots of BID, tBID (truncated BID), BIM and PUMA in PMs and analysis results (n = 5). **(D-E)** Coimmunoprecipitation (Co-IP) of activated Casp4 binds to BID in HEK293T cells (n = 3). HEK293T cells were transfected with the indicated plasmids for 24 h and the lysates incubated with anti-Flag-magnetic-beads, then immunoblotted with antibodies against Flag or HA. **(F)** Representative immunoblots of BID, tBID, Casp3, and Cl-Casp3 in PMs and analysis results (n = 6). **(G)** Representative immunoblots of BID, tBID, Casp3, and Cl-Casp3 in RAW264.7 cells and analysis results (n = 5). **(H)** Representative immunofluorescence staining sections from mice after 7 d of the injury for BID (light gray), F4/80 (green), and Casp11 (red) (n = 7). Nuclei were stained with DAPI (blue). Scale bars: 100 μm. **A**, **B**,** C**,** F** (tBID) and** G** (tBID) were tested using a One-way ANOVA test; **F** (Cl-Casp3) and **G** (Cl-Casp3) were tested using a Welch's ANOVA test.

Further, we investigated whether the Casp9-Casp3 or Casp8-Casp3 pathway contributed to the GSDME activation. We observed that GA2 triggered the cleaved-Casp9 (Cl-Casp9) and cleaved-Casp3 (Cl-Casp3) formation, not cleaved-Casp8 (Cl-Casp8) (Figure 6A). We also found that GA2 triggered the leakage of Cyt C to the cytosol (Cyto), which regulated Cl-Casp9 formation (Figure 6B). We then tested the key members of BH3-only proteins, which regulate the release of cytochrome C to the cytosol. We found that GA2 triggered the formation of truncated BID (tBID), but did not influence the expression of BIM and PUMA (Figure 6C). These findings were also confirmed in HMDMs and RAW264.7 cells, indicating that GA2 induced the BID formation and the Casp3 activation (Figure S4). We speculated that the activated Casp4/11 cleaved BID to tBID, which regulated the leakage of Cyt C into the cytosol. A recent study revealed that auto-processing at Casp4 Asp289, generating the p33/p10 form combined with substrate, could be detected 29. Similarly, to capture the binding signal between Casp4 and BID, protease-deficient Casp4 (C258A) mutant plasmids were used, including the full-length fragment, the p33 fragment containing the CARD domain and protease domain, and the p22 fragment containing only the protease domain. In HEK293T cells, the full length of Casp4, p33, or p22 was not co-immunoprecipitated with BID (Figure 6D). Surprisingly, an interaction between the Casp4 p33/p10 form and BID was observed in HEK293T cells but not in the p22/p10 form (Figure 6E). Further, Casp11 deficiency blocked the expression of tBID and Cl-Casp3 in PMs and RAW264.7 cells (Figure 6F and 6G). The co-localization of Casp11, F4/80, and BID increased in WT mice stimulated with GA2 7 days after injury (Figure 6H). Collectively, these results suggested that GA2-mediated Casp4/11 activation contributed to tBID-mediated Cytochrome C-Casp9-Casp3-GSDME pathway activation.

**Figure 7:**
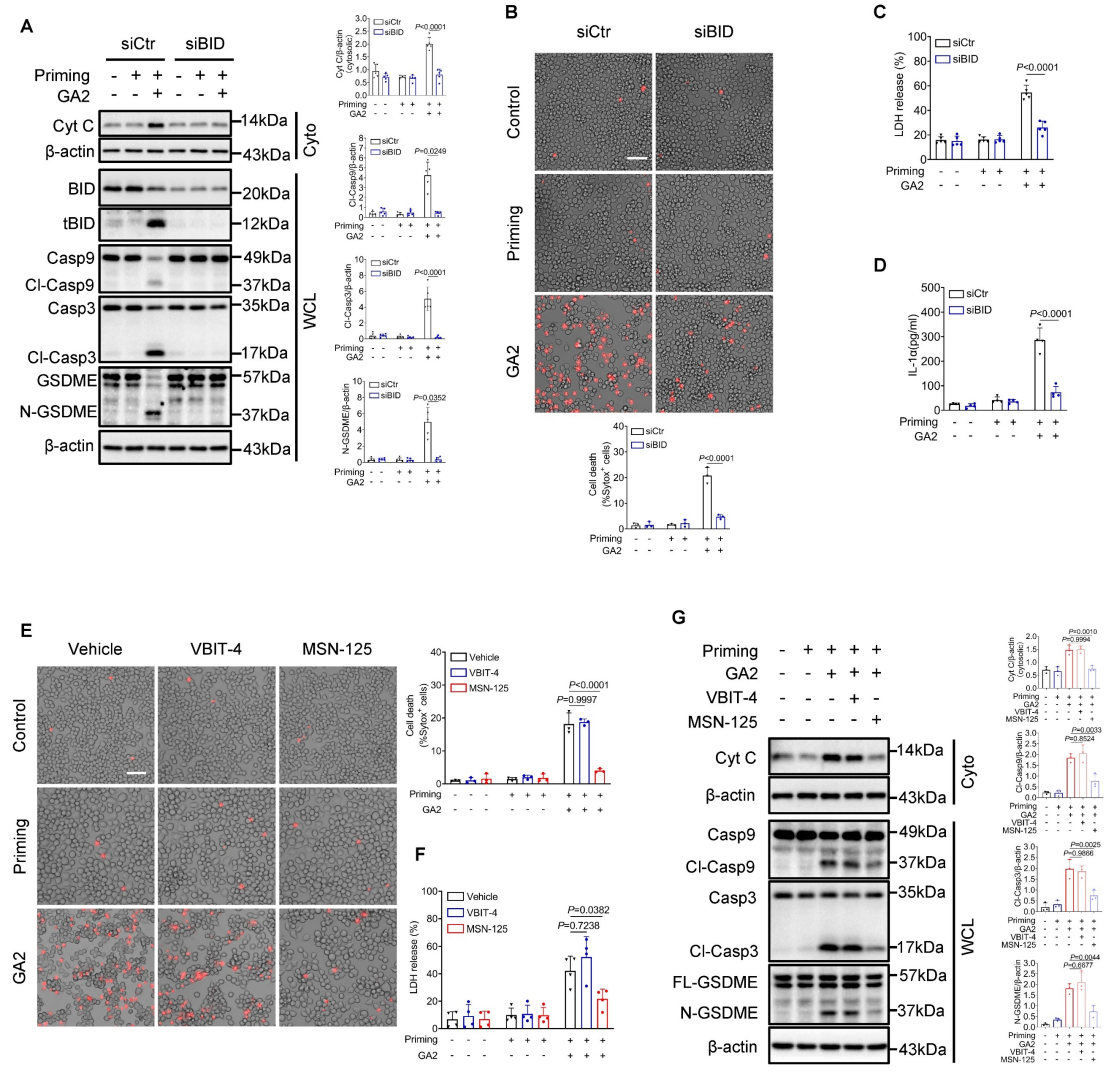
** The Knock-down of BID and pharmacological inhibition of Bax/Bak prevents macrophage pyroptosis. (A)** Representative immunoblots and analysis results of Cyt C in cytosol (Cyto), BID, tBID, Casp9, Cl-Casp9, Casp3, Cl-Casp3, GSDME, and N-GSDME in whole cell lysates (WCL) (n = 5). RAW264.7 cells were transfected with siBID (siRNAs targeting BID) or siCtr for 30 h, and then treated as indicated. **(B)** Cell death of RAW264.7 cells (n = 3). Scale bars: 50 μm. **(C)** LDH release of RAW264.7 cells (n = 5). **(D)** IL-1α secretion of RAW264.7 cells (n = 4). **(E)** Cell death of RAW264.7 cells (n = 3). Cells were primed or not primed with 100 ng/mL LPS for 6 h, and pretreated with 1 μM VBIT-4, 1 μM MSN-125 or equimolar DMSO (vehicle) for 30 min, and then treated as indicated.** (F)** LDH release of RAW264.7 cells (n = 5). **(G)** Representative immunoblots and and analysis results of Cyt C in Cyto, Casp9, Cl-Casp9, Casp3, Cl-Casp3, GSDME, and N-GSDME in WCL (n = 3). **A** (Cyt C and Cl-Casp3), **B**,** C**,** D, E, F** and** G** were tested using a One-way ANOVA test; **A** (Cl-Casp9 and N-GSDME) were tested using a Welch's ANOVA test.

Moreover, small interfering RNA to knock down BID (siBID) significantly inhibited the expression of cytosolic Cyt C and the expression of Cl-Casp9, Cl-Casp3, and N-GSDME in RAW264.7 cells treated with GA2 (Figure 7A). SiBID significantly decreased cell death, LDH release, and the secretion of IL-1α (Figure 7B-7D). BAX and BAK are known as the executors of tBID, triggering the release of mitochondrial cytochrome C into the cytosol 30, 31. Voltage-dependent anion channel-1 (VDAC1) also regulates the leakage of mitochondrial cytochrome C 32. MSN-125 and VBIT4 are specific inhibitors of Bax/Bak oligomerization and VBIT4 oligomerization, respectively 33, 34. As expected, the pharmacological inhibition of Bax/Bak, not VDAC1, significantly decreased cell death, LDH release, cytosolic Cyt C expression, and the expression of Cl-Casp9, Cl-Casp3, and N-GSDME induced by GA2 in RAW264.7 cells (Figure 7E-7G).

### Transplantation of Casp11^-/-^ bone marrow into WT mice or specific knockdown of Casp11 in bone marrow-derived macrophages attenuates GA2-induced exacerbation of IH

**Figure 8:**
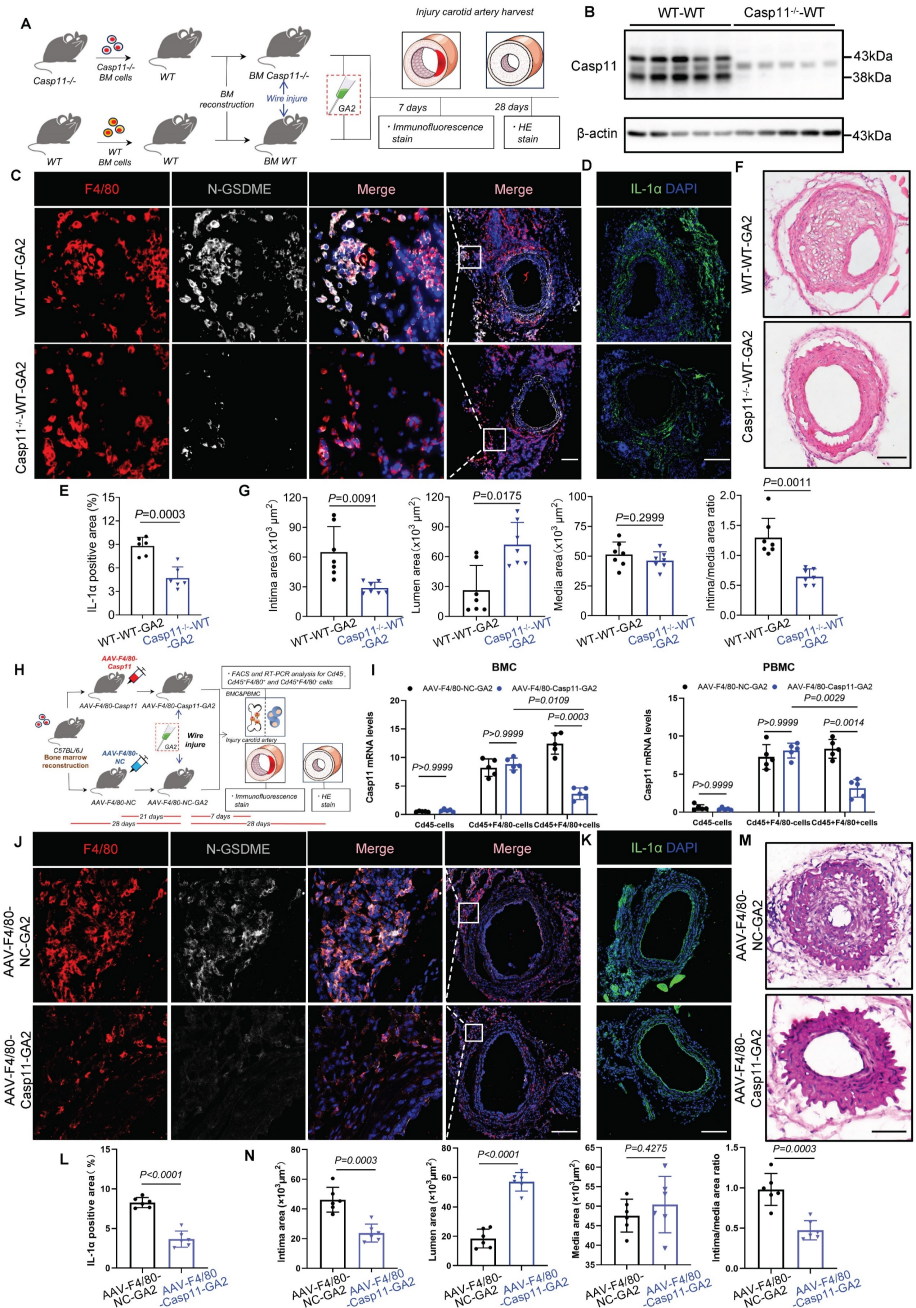
** Transplanted with Casp11^-/-^ bone marrow or specific knockdown of Casp11 in bone marrow-derived macrophages protects against ganglioside GA2-induced exacerbation of IH. (A)** Experimental design for bone marrow transplantation and intervention. **(B)** Immunoblots for analyzing the Casp11 expression of bone marrow-derived myeloid cells from chimeric mice. WT mice were transplanted with Casp11^-/-^ (Casp11^-/-^-WT) or WT (WT-WT) bone marrow. **(C)** Representative immunofluorescence staining sections from mice after 7 d of the injury for F4/80 (red) and N-GSDME (light gray) (n = 6). Nuclei were stained with DAPI (blue). Scale bars: 100 μm. **(D-E)** Representative immunofluorescence staining sections from mice after 7 d of the injury for IL-1α (green) and quantitative analysis. Nuclei were stained with DAPI (blue) (n = 6). Scale bars: 100 μm. **(F-G)** Representative HE-stained sections from mice after 28 d of the injury, and analysis results of intima area, lumen area, media area, and intima/media area ratio (n = 7). Scale bars: 100 μm. **(H)** Experimental design for bone marrow macrophage specific Casp11-knockdown and intervention. **(I)** RT-qPCR analysis of Casp11 mRNA expression in Cd45^+^F4/80^+^ cells and Cd45^+^F4/80^-^ cells from mice (n = 5). Single-cell suspensions isolated from bone marrow cells (BMC) and Peripheral blood mononuclear cells (PBMC) of mice. WT mice were treated either with vehicle or GA2 through tail intravenous injection one day before surgery and every other day after surgery for 7 d among AAV-F4/80-NC group (AAV-F4/80-NC-GA2) and AAV-F4/80-Casp11 group (AAV-F4/80-Casp11-GA2). **(J)** Representative immunofluorescence staining sections from AAV-F4/80-NC-GA2 and AAV-F4/80-Casp11-GA2 after 7 d of the injury for F4/80 (red) and N-GSDME (light gray) (n = 6). Nuclei were stained with DAPI (blue). Scale bars: 100 μm. **(K-L)** Representative immunofluorescence staining sections from mice after 7 d of the injury for IL-1α (green) and quantitative analysis. Nuclei were stained with DAPI (blue) (n = 6). Scale bars: 100 μm. **(M-N)** Representative HE-stained sections from mice after 28 d of the injury, and analysis results of intima area, lumen area, media area, and intima/media area ratio among AAV-F4/80-NC-GA2 and AAV-F4/80-Caspase11-GA2 groups (n = 6). Scale bars: 100 μm. **E**,** G** (Media), **I**,** L** and **N** were tested using a two-tailed Student's *t*-test; **G** (Intima and Intima/media) were tested using a two-tailed Welch's *t*-test; **G** (Lemun) was tested using a two-tailed Mann-Whitney *U* test.

Transplanting the bone marrow of genetically deficient mice is an effective method to knock out a gene in myeloid macrophages 17, 35. Having detected that Casp11 contributed to GA2-induced macrophage pyroptosis, we next used bone marrow transplantations to evaluate the effect of Casp11 on IH induced by GA2 (Figure 8A). Bone marrow-derived myeloid cells lack Casp11 expressions in the chimeric mice (Figure 8B). It was observed that the expression of N-GSDME expression in macrophages and the expression area of IL-1α were decreased in Casp11^-/-^-WT chimeric mice stimulated with GA2 at 7 days after injury, compared with WT mice transplanted with WT bone marrow (Figure 8C-8E). Prevention of IH was observed in WT mice transplanted with Casp11^-/-^ bone marrow at 28 days after carotid arterial injury, as indicated by a decrease in intimal area and the intima/media area ratio and an increase in lumen area (Figure 8F and 8G).

To further improve macrophage specificity, an F4/80 gene promoter was used to construct an adeno-associated virus 2/9 carrying the encoding Casp11 shRNA (AAV-F4/80-Casp11), which was injected intravascularly 7 days after bone marrow transplant. At 21 days after AAV-F4/80-Casp11 injection, carotid artery injury models were applied (Figure 8H). The specificity and efficacy of Casp11-knockdown in macrophages (CD45+F4/80+) of PBMCs and BMCs were confirmed through FACS and RT-qPCR analysis (Figure 8I). It was observed that the expression of N-GSDME in macrophages and the expression area of IL-1α decreased in AAV-F4/80-Casp11-GA2 mice 7 days after injury, compared with AAV-F4/80-NC-GA2 mice (Figure 8J-8L). A prevention of IH was observed in AAV-F4/80-Casp11-GA2 mice 28 days after carotid arterial injury (Figure 8M and 8N). This indicated that the specific knockdown of Casp11 in bone marrow-derived macrophages improves the deterioration of IH caused by GA2.

## Discussion

Here, we have demonstrated for the first time that the GA2-Casp11 pathway contributed to IH. First, we identified that GA2 was remarkably accumulated in both the artery and plasma of atherosclerotic patients and mice. GA2 aggravated IH and co-stained with macrophages after mouse carotid arterial injury. GA2 was internalized into PMs through caveolin-1 *in vitro*. The internalization of GA2 triggered the Casp4/11 activation, N-GSDME-mediated pyroptosis, and IL-1α release. Deletion of Casp11 abolished the N-GSDME-mediated pyroptosis and IL-1α secretion in macrophages. Mechanistically, GA2 directly combined with Casp4 and induced Casp4 activation. Then, activated Casp4/11 combined with and cleaved BID, promoting the release of cytochrome C to the cytoplasm. Cytoplasmic cytochrome C drove GSDME-mediated pyroptosis through the activation of the Casp9-Casp3 pathway. Finally, in the carotid arterial injury model in mice, we demonstrated that transplantation with Casp11-deficient bone marrow rescued the IH exacerbated by GA2. Collectively, these findings suggest a potential diagnostic and therapeutic target for IH.

Here, we identified that GA2 was accumulated in the aortae and plasma of atherosclerotic mice. GA2 was also upregulated in the atherosclerotic plaque of the human thoracic aorta and plasma of patients with CHD. Injection of GA2 aggravated IH in mouse model. The co-stained of GA2 and macrophages was observed at the arterial injury site and atherosclerotic plaque of the human thoracic aorta. It has been reported that caveolin-1 was unregulated plaques and promoted lipid accumulation in atherosclerosis 36-39. Glycosphingolipids are uptake by cells dependent on caveolin-1-mediated phagocytosis 21, 22. We also found that GA2 was internalized into PMs through caveolin-1 *in vitro*. Therefore, caveolin-1 could promote the accumulation of GA2 in atherosclerotic aortae and IH. Further, we observed that internalization of GA2 induced the cell death of mouse inflammatory macrophages through triggered Casp4/11-GSDME activation. This mode of cell death was defined as pyroptosis. Knockout Casp11 blocked GSDME activation and macrophage pyroptosis. The most typical molecular mechanism for Casp4/11 activation is that Casp4/11 recognizes and interacts with cytoplasmic danger molecular, then auto-activates 11, 12, 40. We found that GA2 directly combined with Casp4 and elicited its activation. Meanwhile, we analyzed the contribution of the oligosaccharide headgroup and ceramide residue of GA2 to Casp4 activation 1. Both of them did not trigger Casp4 activation. These results supported that the oligosaccharide headgroup and ceramide residue were necessary for GA2 to activate Casp4. Studies reported that the polar glycan headgroup and fatty acid chains of lipid A could be directly detected by Casp4/11 and then trigger Casp4/11 auto-activation 11, 12. The structure of GA2 is similar to lipid A in that it contains the NAc-oligosaccharide headgroup (polar glycan headgroup) and ceramide residue (fatty acid chains) 1, 16. Hence, our results suggest that cytoplasmic GA2 induced the activation of Casp4/11 as a ligand.

It is firmly established that the activation of Casp9-Casp3 or Casp8-Casp3 induces N-GSDME formation and pyroptosis 41-43. Our findings presented that GA2 triggered Casp9-Casp3 activation but not Casp8. Further, we observed that the release of cytochrome C from mitochondria to cytosol is the critical process for Casp9 activation 43, 44. Moreover, GA2 significantly increased the expression of tBID. Studies reported that BID is activated as a substrate of different caspases 45-49. Our findings show that the activated Casp4 p33/p10 form co-immunoprecipitated with BID in HEK293T cells. Knock-down BID significantly inhibited the cytochrome C-Casp9-Casp3-GSDME pathway activation, thereby reducing macrophage pyroptosis. Additionally, the pharmacological inhibition of Bax/Bak further confirmed that tBID induced the release of mitochondrial cytochrome C through BAX/BAK, activating downstream signals. Our findings suggested that GA2-Casp11-mediated BID-Casp9-Casp3-GSDME signal activation contributed to macrophage pyroptosis. Previous studies have indicated that Casp4/11 activated by different stimulation may select diverse substrates 24, 27, 40. Our study showed cleaved-Casp4/11 can trigger tBID formation in GA2 treatment. The specific mechanisms warrant further investigation. A recent study indicated that mitochondrial permeability transition driving the Apaf-1/Casp4 pryoptosome formation triggered pyroptosis 27. Differently, in our experiments, Casp4/11 was first activated by GA2, and then tBID formation was induced to trigger mitochondrial dysfunction. Both manners induced GSDME activation and macrophage pyroptosis. These suggested that Casp4/11 may be a key regulator in the relationship between mitochondria and cell fate.

IL-1s, including IL-1α and IL-1β, are potent proinflammatory factors regulating smooth muscle cell proliferation and migration, leukocyte adherence, and extracellular matrix production and remodeling 8, 9. A large number of early studies have demonstrated the key role of IL-1 in IH 7, 50, 51. In our experiment, we found that GA2-induced Casp11 activation driven N-GSDME-mediated macrophage pyroptosis and accompanied the release of cytokine IL-1α. In the mice carotid arterial injury model, we confirmed that an increasing number of the N-GSDME expression in macrophages and the expression area of IL-1α were triggered by GA2 stimulation. Further, GA2 exposure exacerbated the IH after carotid arterial injury. Furthermore, Casp11^-/-^ bone marrow transplantations attenuated the IH, the number of the N-GSDME expression in macrophages, and the expression area of IL-1α. More importantly, Casp11 knockdown in BM macrophages alleviated the IH, the expression of N-GSDME in macrophages and the expression area of IL-1α in mice performed with wire injury followed by GA2 injection. Collectively, these findings supported that GA2 triggered caspase-11-mediated macrophage pyroptosis and the release of cytokine IL-1α to aggravate IH.

Overall, for the first time to our knowledge, our findings showed that GA2-regulated Casp4/11 signal controls tBID-mediated Cytochrome C-Casp9-Casp3-GSDME pathway activation, which further triggers macrophage pyroptosis and IL-1α release to accelerate the IH after arterial injury. These findings suggest that GA2-Casp11 pathway-mediated macrophage pyroptosis may represent a new diagnostic and therapeutic strategy for IH.

## Supplementary Material

Supplementary figures and table.

Supplementary lipidomics concentration.

Supplementary class concentration.

Supplementary CerG2GNAc1 concentration in mouse plasma.
