# Supplementary material for: Ganglioside GA2-mediated caspase-11 activation drives macrophage pyroptosis aggravating intimal hyperplasia after arterial injury
Source: Int J Biol Sci. 2025 Jan 1;21(1):433–53. doi: 10.7150/ijbs.97106 (PMC11667815; doi:10.7150/ijbs.97106)
Supplement: Supplementary file 1 — Supplementary figures and table. [file ijbsv21p0433s1.pdf]

Ganglioside GA2-mediated caspase-11 activation drives macrophage pyroptosis aggravating intimal hyperplasia after arterial injury

Yunmin Shi[1,2#], Tian He[1#], Hong Liu[1,3], Xiaodong Li[4], Zhengxin Li[1], Qing Wen[1], Zhaohui Dai[5], Xuejing Sun[1], Qian Tan[1], Wenjing Yang[1], Youxiang Jiang[1], Yuanyuan Liu[1], Hong Yuan[1], Fang Lei[6*], Yang Yi[7*], Jingjing Cai[1*]

[1] Department of Cardiology, Third Xiangya Hospital, Central South University, Changsha, 410013, Hunan, China

[2] Cancer Institute (Key Laboratory of Cancer Prevention and Intervention, China National Ministry of Education), The Second Affiliated Hospital, Zhejiang University School of Medicine, Hangzhou, 310009, China

[3] Research Center for Life Science and Human Health, Binjiang Institute of Zhejiang University, Hangzhou, 310053, China

[4] Department of Cardiovascular Medicine, Department of Hypertension, Ruijin Hospital and State Key Laboratory of Medical Genomics, Shanghai Key Laboratory of Hypertension, Shanghai Institute of Hypertension, Shanghai Jiao Tong University School of Medicine, 200020, China.

[5] Department of Cardiology of the Fourth Hospital of Changsha, Changsha Hospital of Hunan Normal University, Changsha, 410006, Hunan, China

[6] Medical Science Research Center, Zhongnan Hospital of Wuhan University, Wuhan, 430000, Hubei, China

[7] Department of Cardiovascular Surgery, Ruijin Hospital, Shanghai Jiao Tong University School of Medicine, 200020, Shanghai, China

[#]These authors contributed equally to this work.

[*]Corresponding Author

Supplementary Table

Table S1. Demographic and baseline characteristics of the patients.

Parameter	Total (n=16)	CTL (n=8)	CHD (n=8)	P-value
Age,median(IQR),years	61.0(44.5-69.5)	57.5(44.5-69.5)	64.5(62.0-68.0)	0.4210
Men,n(%)	9(56.2)	4(50)	5(62.5)	>0.9999
SBP,mean±SD,mmHg	130.20±20.27	132.80±24.21	127.6±16.70	0.6298
DBP,mean±SD,mmHg	78.75±13.07	83.50±14.02	74.00±10.86	0.1520
HR,mean±SD,beats/min	75.19±8.57	79.00±9.957	71.38±4.984	0.0732
**Laboratory Findings**				
WBC,median(IQR),*10^9/L	6.43(5.10-7.83)	6.84(5.28-8.35)	6.01(5.10-6.43)	0.3669
PLT,mean±SD,*10^9/L	180.40±53.11	153.0±44.81	207.8±48.20	0.0338
FBG,mean±SD,mmol/L	5.04±0.59	4.913±0.4486	5.175±0.6541	0.3651
ALT,mean±SD,U/L	27.19±14.54	22.63±13.63	31.75±14.83	0.2208
Cr,mean±SD,μmol/L	85.29±27.34	73.88±25.36	96.71±25.70	0.0952
LDL-C,mean±SD,μmol/L	2.35±0.48	2.231±0.4843	2.468±0.4690	0.3384
**Baseline medications, n (%)**				
ACEI/ARB	9(56.25)	5(62.50)	4(50.00)	>0.9999
CCB	6(37.50)	3(37.50)	3(37.50)	>0.9999
β-blocker	10(62.50)	3(37.50)	7(87.50)	0.1189
Antiplatelets	3(18.75)	0(0.00)	3(37.50)	0.2000
P2Y12 inhibition	5(31.25)	0(0.00)	5(62.50)	0.0256
Statins	12(75.00)	4(50.00)	8(100.00)	0.0769
**Comorbidities, n (%)**				
Diabetes	0(0.00)	0(0.00)	0(0.00)	-
Hypertension	7(43.75)	3(37.50)	4(50.00)	>0.9999
Atrial fibrillation	0(0.00)	0(0.00)	0(0.00)	-
Stroke	0(0.00)	0(0.00)	0(0.00)	-

Values are mean ± SD, median (IQR) or n (%). SD: standard deviation; IQR: interquartile range; SBP: systolic blood pressure; DBP: diastolic blood pressure; HR: heart rate; WBC: white blood cell; PLT: platelet; FBG: fast blood glucose; ALT: alanine transaminase; Cr: creatinine; LDL-C: low-density lipoprotein cholesterol; ACEI/ARB: angiotensin-converting enzyme inhibitor or angiotensin receptor blocker; CCB: calcium-channel blocker; β-blocker: β-receptor blocker. P2Y12 inhibition: clopidogrel, prasugrel, ticagrelor.

Supplementary Figures

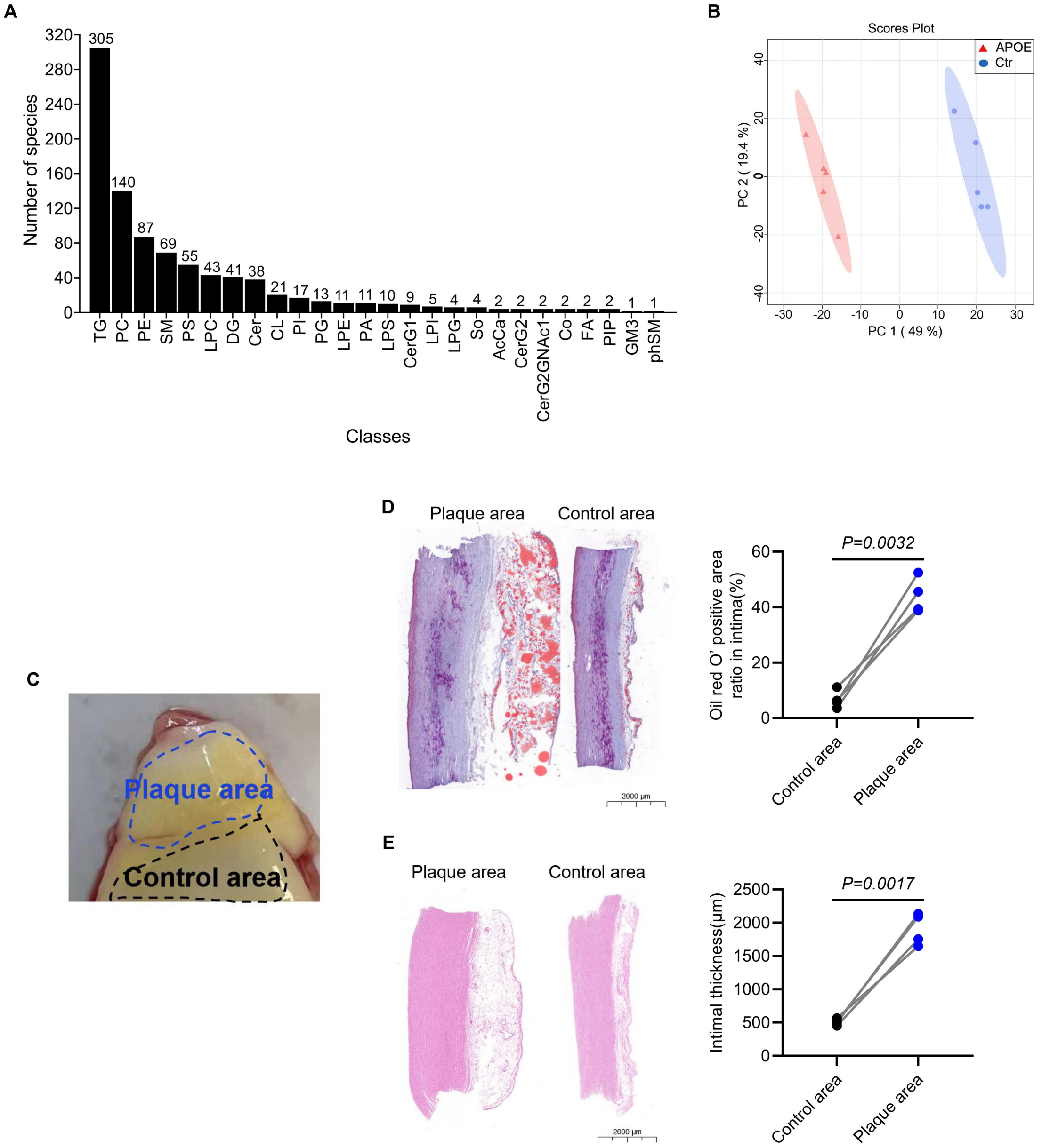

Figure S1. (A) Total lipidomic classes and species were identifed in mouse aortae. **(B)** Scores scatter plot corresponding to a principal component analysis of the Lipidomics concentration in the aortae of mice (n = 5). Wild type (WT) mice fed a normal laboratory diet for 16 weeks served as control (Ctr) group. APOE$^{-/-}$ mice fed high-fat high-cholesterol diet for 16 weeks served as APOE group. **(C)** Representative image of atherosclerotic lesions in the human thoracic aorta. **(D)** Oil-red O staining in plaque and non-plaque areas in human arteries (N=4 patients). Scale bars: 2 mm. **(E)** Intimal thickness by hematoxylin and eosin-staining (H&E) in plaque and non-plaque areas in human arteries (N=4 patients). Scale

bars: 2 mm. **D** and **E** was tested using a two-tailed paired *t*-test.

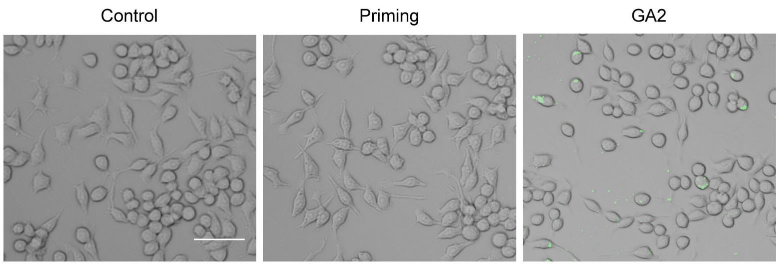

Figure S2. The endocytosis of ganglioside GA2 in RAW264.7 cells.

The endocytosis of ganglioside GA2 (GA2) by inflammatory macrophages (n = 3). Green indicates the GA2. Priming cells were treated with 4 μg/mL fluorescent probe labeled-GA2 for 8 h. Priming or non-priming cells stimulated with equimolar DMSO were set as a priming group or a control group. Scale bars: 50 μm

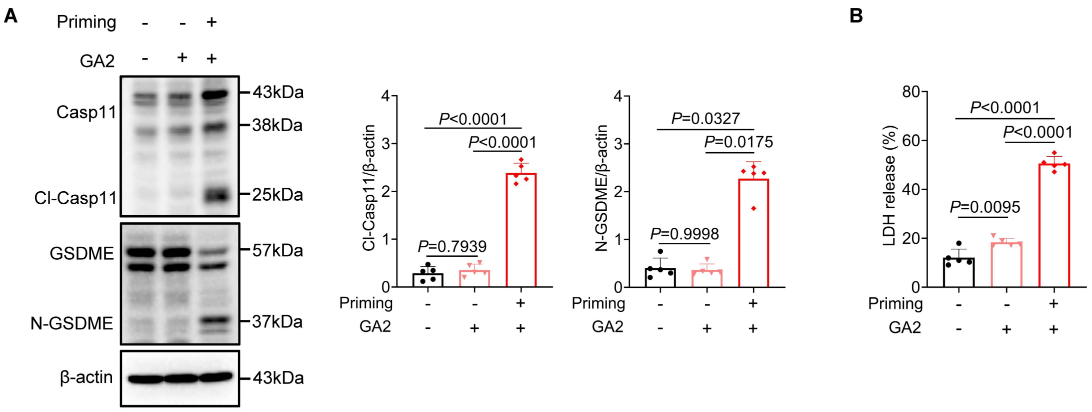

Figure S3. GA2 triggers pyroptosis in the priming mouse macrophages.

(A) Representative immunoblots of Casp11, Cl-Casp11, GSDME and N-GSDME in RAW264.7 cells and analysis results (n = 5). Priming or non-priming cells were transfected with 6 μg GA2 for 20 h. Non-priming cells transfected with equimolar DMSO were set as a control group. **(B)** LDH release of RAW264.7 cells (n = 5). **A** (Cl-Casp11) and **B** were tested using a One-way ANOVA test; **A** (N-GSDME) was tested using a Kruskal Wallis test.

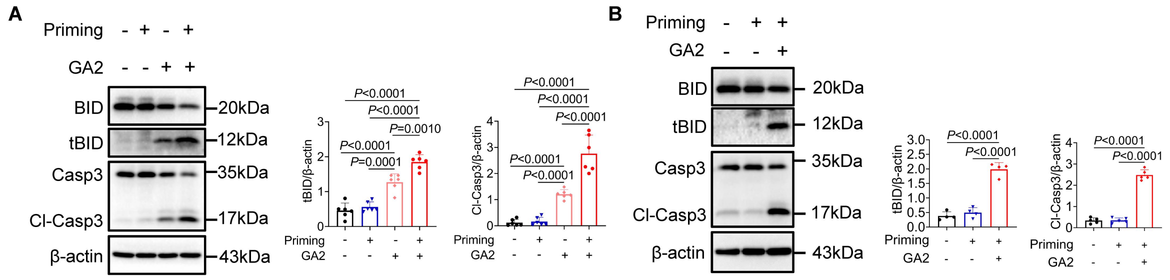

Figure S4. GA2 induces Casp3 and BID activation in macrophages.

(A) Representative immunoblots of BID, tBID, Casp3 and Cl-Casp3 in HMDM cell lysates and analysis results (n = 6). **(B)** Representative immunoblots of BID, tBID, Casp3 and Cl-Casp3 in RAW264.7 cells and analysis results (n = 5). **A** (tBID) and **B** (Cl-Casp3) were tested using a One-way ANOVA test; **A** (Cl-Casp3) and **B** (tBID) were tested using a Welch's ANOVA test.
